# Prediction of Thorough QT study results using action potential simulations based on ion channel screens

**DOI:** 10.1016/j.vascn.2014.07.002

**Published:** 2014-11

**Authors:** Gary R. Mirams, Mark R. Davies, Stephen J. Brough, Matthew H. Bridgland-Taylor, Yi Cui, David J. Gavaghan, Najah Abi-Gerges

**Affiliations:** a Computational Biology, Dept. of Computer Science, University of Oxford, Oxford OX1 3QD, UK; b Clinical Informatics, R&D Information, AstraZeneca, Alderley Park, SK10 4TG, UK; c Screening & Compound Profiling, GlaxoSmithKline, Stevenage SG1 2NY, UK; d Discovery Sciences, AstraZeneca, Alderley Park, SK10 4TG, UK; e Safety Evaluation and Risk Management, Global Clinical Safety, GlaxoSmithKline, Middlesex UB11 1BT, UK; f Translational Safety Department, Drug Safety & Metabolism, AstraZeneca, Alderley Park, SK10 4TG, UK

**Keywords:** AP(D), action potential (duration), AZ, AstraZeneca, B&Q2, IonWorks Barracuda and second Quattro screening dataset, GLP, good laboratory practice, GSK, GlaxoSmithKline, IC_50_, concentration for 50% inhibition, I_CaL_, long-lasting-type calcium current, I_Kr_, rapid delayed rectifier potassium current, I_Ks_, slow delayed rectifier potassium current, I_Na_, fast sodium current, I_to_, transient outward potassium current, M&Q, manual hERG plus IonWorks Quattro screening dataset, pIC_50_, minus log_10_ of IC_50_, Q, IonWorks Quattro screening dataset, QT[c], QT interval of the electrocardiogram [corrected for heart rate], TQT, Thorough QT (or ECG) study, a human clinical trial, High-throughput, Compound screening, Thorough QT, Cardiac safety, Methods, Action potential, Mathematical model

## Abstract

**Introduction:**

Detection of drug-induced pro-arrhythmic risk is a primary concern for pharmaceutical companies and regulators. Increased risk is linked to prolongation of the QT interval on the body surface ECG. Recent studies have shown that multiple ion channel interactions can be required to predict changes in ventricular repolarisation and therefore QT intervals. In this study we attempt to predict the result of the human clinical Thorough QT (TQT) study, using multiple ion channel screening which is available early in drug development.

**Methods:**

Ion current reduction was measured, in the presence of marketed drugs which have had a TQT study, for channels encoded by hERG, CaV1.2, NaV1.5, KCNQ1/MinK, and Kv4.3/KChIP2.2. The screen was performed on two platforms — IonWorks Quattro (all 5 channels, 34 compounds), and IonWorks Barracuda (hERG & CaV1.2, 26 compounds). Concentration–effect curves were fitted to the resulting data, and used to calculate a percentage reduction in each current at a given concentration.

Action potential simulations were then performed using the ten Tusscher and Panfilov (2006), Grandi et al. (2010) and O'Hara et al. (2011) human ventricular action potential models, pacing at 1 Hz and running to steady state, for a range of concentrations.

**Results:**

We compared simulated action potential duration predictions with the QT prolongation observed in the TQT studies. At the estimated concentrations, simulations tended to underestimate any observed QT prolongation. When considering a wider range of concentrations, and conventional patch clamp rather than screening data for hERG, prolongation of ≥ 5 ms was predicted with up to 79% sensitivity and 100% specificity.

**Discussion:**

This study provides a proof-of-principle for the prediction of human TQT study results using data available early in drug development. We highlight a number of areas that need refinement to improve the method's predictive power, but the results suggest that such approaches will provide a useful tool in cardiac safety assessment.

## 1 Introduction

The problem of drug-induced pro-arrhythmic risk is now well recognised, and substantial resources are currently allocated to assessing this risk throughout drug development (Pollard et al., 2010). This begins with the assessment of a new compound's affinity for blocking the current carried by the hERG channel (ICH-S7B, 2005, Redfern et al., 2003), typically including in-vitro/ex-vivo animal-based models at mid-stage safety testing, before in-vivo assessment in a number of species in late pre-clinical safety testing (Carlsson, 2006).

At present, the definitive assessment of clinical risk is usually considered to be provided by the human clinical Phase II/III Thorough QT [or ECG] (TQT) study, as recommended by the ICH (2005) guidelines. Block of hERG has long been associated with prolongation of ventricular repolarisation and increased pro-arrhythmic risk (Sanguinetti & Tristani-Firouzi, 2006). The value of hERG 50% inhibitory concentrations (IC_50_s) for predicting TQT results was assessed by Gintant (2011): using a safety margin value of 45 (free plasma concentration should be 45 times smaller than IC_50_) was 64% sensitive and 88% specific for TQT prolongation of ≥ 5 ms.

It has been suggested that multiple-ion-channel effects should be considered to provide a more accurate assessment of pro-arrhythmic risk (Kramer et al., 2013, Mirams et al., 2011), and that simulations based on mathematical models for the electrophysiology of cardiac myocytes could be used to integrate information on how a compound affects different ion channels (Fletcher et al., 2011, Gintant, 2012, Mirams et al., 2012, Mirams and Noble, 2011).

A recent Comprehensive in-vitro Pro-arrhythmia Assay (CiPA) initiative led by the US Food & Drug Administration, the Cardiac Safety Research Consortium (www.cardiac-safety.org), the Health and Environmental Sciences Institute (www.hesiglobal.org), and the Safety Pharmacology Society (http://safetypharmacology.org) aims to use this type of approach to provide accurate mechanistic predictions of pro-arrhythmic risk (Sager, Gintant, Turner, Pettit, & Stockbridge, 2014). In this study we aim to evaluate how well action potential simulations, based upon cardiac ion channel screening data, could predict the result of the TQT study. In doing so, we provide a feasibility study for the in-silico aspects of the CiPA initiative, and highlight some issues that are going to be important for its success.

## 2 Methods

**Fig. 1:**
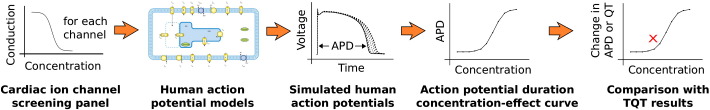
An overview of the steps involved in this study. Ion channel concentration-effect data are taken from a number of screening sources, then used to calculate percentage reduction for the parameters describing maximum conductance of the currents in a human in-silico action potential model. Steady pacing at 1Hz is used to simulate an APD_90_ comparable with QTc. The process is repeated across a range of concentrations, and compared with the TQT study result at the relevant estimated concentration. The various steps are discussed in Methods 2.1, 2.2, 2.3, 2.4, 2.5.

An overview of the procedure used in this study is shown in Fig. 1, and we outline the steps in the sections below.

### 2.1 Screening

A methods description for the IonWorks Quattro screening performed at AstraZeneca (AZ) on all five channels, for 34 compounds, can be found in Elkins et al. (2013) and Supplementary Material S1.2.1. We refer to this dataset as the Quattro (Q) dataset. A methods description for a second screening performed at GlaxoSmithKline (GSK) using IonWorks Barracuda for HERG and CaV1.2 (together with a second Quattro screen for NaV1.5 and KCNQ1) for 26 compounds can be found in Supplementary Material S1.2.2; this is referred to as the Barracuda & second Quattro (B&Q2) dataset. All of the methods descriptions have also been entered into the Minimum Information about a Cardiac Electrophysiology Experiment database (MICEE: www.micee.org, Quinn et al. (2011)).

Compound induced current inhibition is characterised using concentration–effect curves. These curves describe how an ‘effect’ or ‘response’ *R* depends on a ‘dose’ or compound ‘concentration’ [*C*]. In this case, the peak ionic current following a voltage step is recorded repeatedly, and the proportion of peak current that remains after addition of a certain concentration (or *dose*) of a compound is the recorded *effect (or response)*. Such curves are well described by the Hill function (Hill, 1910):

(1) $$R\left(\left[C\right]\right)=\frac{{\left[{\mathrm{IC}}_{50}\right]}^{n}}{{\left[C\right]}^{n}+{\left[{\mathrm{IC}}_{50}\right]}^{n}}={\left[1+{\left(\frac{\left[C\right]}{\left[{\mathrm{IC}}_{50}\right]}\right)}^{n}\right]}^{-1}.$$

Here, [*C*] is the concentration, and there are two parameters: [IC_50_], the half-maximal inhibitory concentration; and the Hill coefficient *n*. In previous work (Beattie et al., 2013, Elkins et al., 2013) we found little benefit, if not just additional uncertainty, in considering the Hill coefficients from these data sources; so in this study we assume that *n* = 1, and fit IC_50_ values only.

### 2.2 Action potential models

We use three of the latest human ventricular action potential models — ten Tusscher and Panfilov (2006), Grandi, Pasqualini, and Bers (2010), and O'Hara, Virág, Varró, and Rudy (2011). These models were chosen as they are all candidates for use in in-silico action potential modelling for cardiac safety, and it will be valuable to examine the consistency of their predictions.

The ten Tusscher and Panfilov (2006) model contains a limited number of differential equations (17) and outer membrane currents (12), and is a refinement of the ten Tusscher, Noble, Noble, and Panfilov (2004) model. The model was developed to provide realistic conduction velocity restitution and to integrate the latest human data at the time. It has been very widely used for a range of studies and has proved robust: making good predictions in a number of situations.

The Grandi model is a human-tailoring of the Shannon, Wang, Puglisi, Weber, and Bers (2004) rabbit model, which features detailed calcium handling. It aimed to improve the balance of repolarizing potassium currents, and to capture reverse-rate dependence of I_Kr_ block. This model is more complex than ten Tusscher, with 14 outer-membrane currents many of which are divided into two for the cleft and bulk sarcolemmal spaces. There are a correspondingly larger number of equations (39).

The O'Hara model is a more recent human ventricular model, much of it was built ‘from scratch’ using data from human hearts. The O'Hara et al. (2011) paper shows improved APD dependence on pacing rate in this model relative to the others. This model has 41 differential equations, again there are 14 types of outer membrane currents, including late sodium.

Having been parameterised by different datasets, these models may represent some of the underlying variation between cells, locations in the heart, or indeed individuals, that could be reflected in the variation observed in the TQT study.

**Fig. 2:**
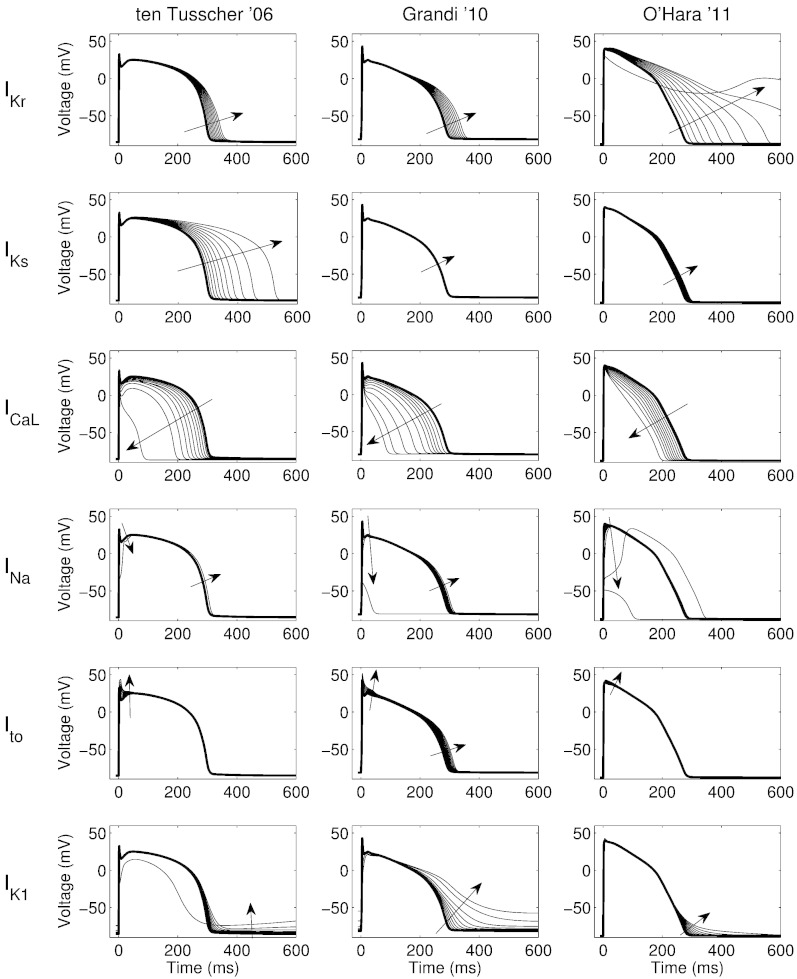
The behaviour of the three human ventricular action potential models used in this study under single current block. Each panel shows the steady state 1 Hz action potential under control (bold line), and increasing degrees of block, from 0% to 100% in steps of 10%. Rows: block of I_Kr_, I_Ks_, I_CaL_, I_Na_, I_to_ or I_K1_; columns: ten Tusscher and Panfilov (2006), Grandi et al. (2010) or O'Hara et al. (2011) models. Arrows indicate the effect on the action potential waveform of increasing channel block.

In Fig. 2 we show basic properties of these models, in terms of response to blockade of certain ion channels, at steady 1 Hz pacing.^1^ Fig. 2 highlights some differences between model behaviours. On the top row we see that the O'Hara model responds more dramatically to block of I_Kr_ than the other models: the action potential becomes markedly prolonged, and at 100% I_Kr_ block the cell fails to repolarise and remains at depolarised potentials. In contrast, the ten Tusscher model shows a large prolongation under I_K__s_ block, whereas the other models show little response. Note that the models may show a larger response under simulated I_Ks_ block in conditions of *β*-adrenergic stimulation (Severi, Corsi, Rocchetti, & Zaza, 2009), which is not included in this study. Under I_K1_ block the models also exhibit a range of responses: the ten Tusscher model resting potential rises to the point that the model becomes self-excitatory and the action potential at 100% block is reminiscent of a stem-cell derived cardiomyocyte or a sino-atrial node cell; the Grandi model shows a large increase in resting potential and also an increase in APD; and the O'Hara model shows a slight increase in APD_90_.

All three models show a shortening of action potential under I_CaL_ block. The largest effect for moderate degrees of I_CaL_ block is observed using the Grandi model. Block of I_Na_ or I_to_ appears to have small effects on action potential duration at up to 80% block in ten Tusscher and O'Hara models, but a small prolongation can occur in both cases in the Grandi model.

### 2.3 Modelling ion channel block

Some early studies have been undertaken to establish binding kinetics for drug interactions with the ion channels (Di Veroli et al., 2012, Moreno et al., 2011). At present these studies are mostly proof-of-principle; we are not aware of any pharmaceutical company parameterising mathematical models of cardiac ion channels and drug kinetics routinely. As a result, we use a conductance-block model for ion channel block, but note that capturing the kinetics of drug/ion channel interaction may become more important in predicting pro-arrhythmia rather than QT prolongation.

The conductance-block model makes a quasi-steady-state approximation for compound binding, and assumes that binding can occur in any channel conformation and that kinetics of channel activity are unaltered after binding (see Brennan, Fink, & Rodriguez, 2009, for a review). Using these approximations, the maximum conductance of a given channel *g*_*j*_, is given by the following function of drug concentration:

(2) $$g_{j}={\left[1+{\left(\frac{\left[C\right]}{\left[{\mathrm{IC}}_{50}\right]}\right)}^{n}\right]}^{-1}{\bar{g}}_{j},$$

where the terms on the right hand side are: the degree of ion-channel block (as given by Eq. (1)) and the maximal conductance of the channel in control conditions (${\bar{g}}_{j}$).

We model block of the following currents:

• I_Kr_ — rapid delayed inward rectifying potassium current; screened using hERG.
• I_Ks_ — slow delayed inward rectifying potassium current; screened using KCNQ1/MinK.
• I_Na_ — fast sodium current; screened using NaV1.5.
• I_CaL_ — long-lasting type calcium current; screened using CaV1.2.
• I_to_ — transient outward potassium current; screened using Kv4.3/KChIP2.2.

These direct relationships between currents and the genes that are over-expressed to screen them are an approximation. The mathematical models of the currents are generally derived from myocyte data, which may include additional ion channels/subunits and regulatory modifications, that the screening cell lines do not possess. For example, in the past, differences were observed between KCNQ1 and I_Ks_ (Silva & Rudy, 2005), and now the MinK subunit is expressed alongside the main channel to produce a more ‘native’ myocyte-like current. Of particular relevance here is the observation that fast I_to_ (Kv4.3) is molecularly distinct from slow I_to_ (Kv1.4) (Niwa & Nerbonne, 2010). Of the three models we consider, O'Hara and ten Tusscher do not include separate fast/slow I_to_ currents, and so the whole current conductance is reduced when applying Eq. (2). The Grandi model does have a distinct fast I_to_ current, and so its conductance is altered directly. Models that have separate I_to_ components may be better for predictions based on screening Kv4.3 in future.

We performed the simulation study three times in parallel, based on the following datasets: Quattro 5 channel (Q); Barracuda & Quattro 4 channel (B&Q2); and a third variant using the Quattro 5 channel screen but with hERG manual patch clamp IC_50_ values replacing the Quattro screening data. The manual data are taken from ICH-S7B Good Laboratory Practice (GLP) studies featured in regulatory submission documents, and gathered by Gintant (2011). We refer to the third dataset as the Manual & Quattro (M&Q) dataset.

### 2.4 Simulation study

Note that QTc is designed to be equal to QT at 1 Hz, so in the simulations we pace cells at 1 Hz (using the square wave stimulus current with magnitude and duration as defined in the models' CellML implementations, see below). We begin with a control simulation, pacing the model until it reaches a pseudo-steady state (see Supplementary Material S1.3 for details on steady state detection). Compound concentration is then increased from 1 nM to 100 μM, taking 20 increments equally spaced on a log_10_ scale. At each concentration, the data shown in Table 1 is used with Eqs. (1), (2) to impose a new maximal conductance value for each of the screened ion currents. We then continue pacing until a new steady state is reached, and evaluate the action potential duration at 90% repolarisation (APD_90_). The process is repeated with all permutations of mathematical model and dataset, giving a total of nine concentration–APD curves per compound.

**Table 1:** All pIC_50_ values (− log_10_ of IC_50_ values in Molar) used for simulation inputs in this study, measured for Quattro (Q), Barracuda (B) as part of this study, and for Manual patch (M) from literature/regulatory submission documents. Q2 refers to a second independent Quattro screening. A zero entry indicates that a pIC_50_ ≤ 0 was fitted to the data, as discussed in the main text.

Compound	hERG			CaV1.2		NaV1.5		KCNQ1		Kv4.3
	Q	B	M	Q	B	Q	Q2	Q	Q2	Q
Alfuzosin	4.7	4.9	3.9	3.7	0.0	3.7	2.8	3.9	0.0	3.0
Alvimopan	1.2	–	3.1	5.3	–	3.6	–	4.3	–	0.0
Ambrisentan	2.1	0.0	3.3	2.6	0.0	2.9	3.0	3.3	4.2	3.6
Darifenacin	5.8	–	7.1	2.8	–	5.8	–	4.7	–	4.9
Darunavir	4.2	4.0	3.8	0.0	2.8	4.4	4.0	3.5	4.3	4.0
Dasatinib	4.3	4.3	4.8	3.4	3.6	4.0	3.4	3.6	0.0	3.5
Deferasirox	0.0	0.0	2.4	3.0	0.0	4.1	3.3	0.0	0.0	4.3
Desvenlafaxine	1.7	0.0	3.6	0.0	0.0	3.7	3.5	0.0	0.0	2.3
Dofetilide	6.9	6.2	8.0	3.8	0.0	3.5	3.2	3.6	3.8	0.0
Doripenem	2.1	–	2.3	5.7	–	2.8	–	3.5	–	0.0
Duloxetine	5.0	5.2	5.3	4.0	4.6	5.1	4.8	5.0	0.0	4.0
Eltrombopag	0.0	2.8	6.2	0.0	0.0	3.8	3.5	0.0	0.0	2.8
Etravirine	3.4	4.6	3.8	0.0	0.0	3.3	2.4	2.9	0.0	2.5
Everolimus	1.8	3.1	3.3	0.0	0.0	3.2	3.7	4.0	0.0	2.0
Lacosamide	0.0	–	1.3	4.3	–	3.3	–	3.6	–	0.0
Lamotrigine	3.4	3.6	3.6	2.8	2.9	4.0	4.4	3.8	0.0	0.0
Lapatinib	1.0	3.2	6.0	2.7	1.8	2.5	0.0	3.6	0.0	0.0
Maraviroc	3.9	4.1	4.4	0.0	0.0	3.0	3.2	4.2	0.0	0.0
Moxifloxacin	3.4	0.0	4.1	3.4	0.0	4.4	3.6	3.8	3.4	0.0
Nebivolol	5.2	5.2	6.5	0.0	4.8	5.2	5.1	4.8	4.4	4.3
Nelfinavir	1.5	3.3	4.9	0.0	0.0	4.1	3.3	4.1	0.0	2.3
Nilotinib	4.2	0.0	6.9	3.7	2.5	3.0	2.3	3.4	0.0	2.5
Paliperidone	6.0	5.9	5.9	3.4	3.0	4.6	3.8	3.6	0.0	4.2
Palonosetron	5.4	–	5.7	3.4	–	4.7	–	4.3	–	0.0
Raltegravir	2.5	3.5	2.8	0.0	0.0	3.5	2.8	4.6	0.0	2.3
Sildenafil	3.8	4.0	4.5	4.0	0.0	3.3	2.9	3.4	3.8	3.3
Silodosin	4.6	–	5.1	3.1	–	4.2	–	3.6	–	3.5
Sitagliptin	3.0	3.3	3.8	1.0	0.0	3.1	3.2	3.5	0.0	1.0
Solifenacin	5.8	–	6.6	5.2	–	5.2	–	4.5	–	4.3
Sunitinib	5.0	5.1	6.6	4.1	3.7	4.8	4.4	4.2	0.0	4.3
Tadalafil	4.1	3.9	4.0	0.0	3.4	3.9	3.8	3.8	3.6	0.0
Telbivudine	2.3	–	0.8	0.0	–	2.5	–	3.6	–	0.0
Tolterodine	6.9	6.8	7.9	0.0	4.6	5.2	4.5	4.1	0.0	4.9
Vardenafil	3.5	4.1	4.5	4.8	3.6	2.6	3.7	3.2	0.0	4.1

**Fig. 3:**
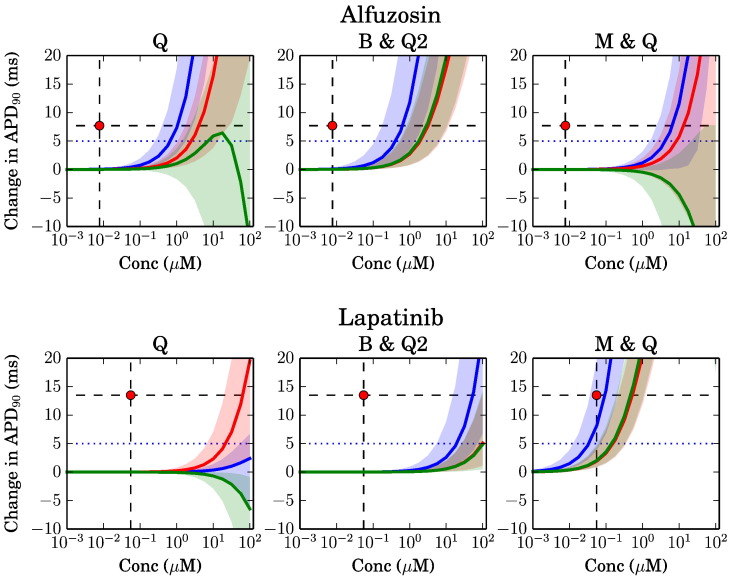
Simulated change in action potential duration (90%) plotted against (free plasma) concentrations. Models: Blue — O'Hara; red — ten Tusscher; green — Grandi. Three data sources are shown for: ‘Q’ (Quattro); ‘B & Q2’ (Barracuda & Quattro); ‘M & Q’ (Manual hERG & Quattro), as per Table 1. Estimated 95% credible regions are shown around each line which capture uncertainty due to screening assay variability. The clinical study result is shown with a black dashed horizontal line for the largest dose in the TQT study; the estimated free plasma concentration associated with this is shown with a vertical dashed black line, and their intersection with a red circle. The 5 ms ‘cut-off’, used in contingency table calculations, is shown with a horizontal blue dotted line.

**Fig. 4:**
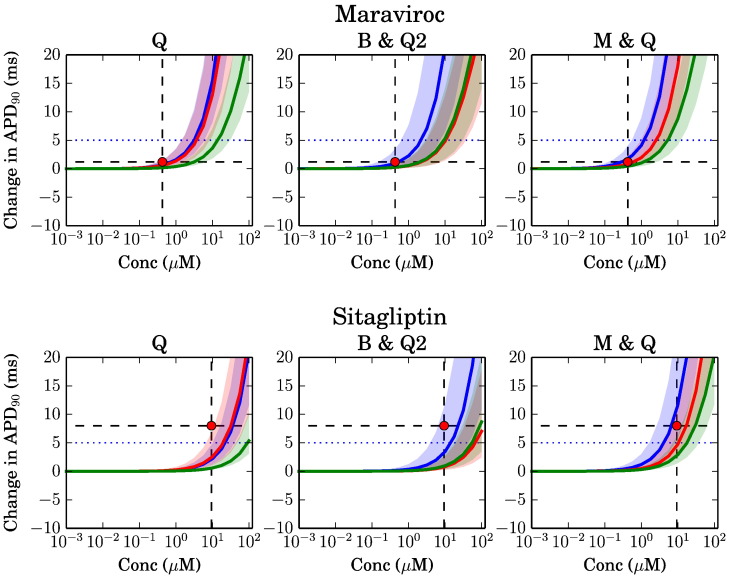
Simulated change in action potential duration (90%) plotted against (free plasma) concentrations. Legend as per Fig. 3.

We use the method outlined in Elkins et al. (2013) to quantify the uncertainty on our APD_90_ predictions due to assay variability. In brief, we characterise variability associated with ion channel screens by examining the pIC_50_ distribution from the relevant control assays. A Bayesian inference scheme then produces a probability distribution for the mean of a large number of independent repeats. pIC_50_ values are then sampled from this distribution at random, and simulations are repeated with these values to build up a distribution of possible outcomes (as displayed in e.g. Fig. 3, Fig. 4). The resulting intervals show where there is 95% probability that the simulation prediction lies, based on the variability we measured in the control screens for each channel.

#### 2.4.1 Software implementation

CellML is a machine-readable XML-based markup language used to describe models' ordinary differential equations, initial conditions and parameters (Lloyd, Lawson, Hunter, & Nielsen, 2008). The ten Tusscher and Panfilov (2006), Grandi et al. (2010), and O'Hara et al. (2011) models were downloaded from the Physiome Project repository (https://models.physiomeproject.org/electrophysiology). The epicardial variants of ten Tusscher and Panfilov (2006) and Grandi et al. (2010) were used, with the endocardial variant of O'Hara et al. (2011) (as this model was primarily parameterised with endocardial data).

PyCML was used to convert the CellML format into C++ code (Cooper, Corrias, Gavaghan, & Noble, 2011). The CellML files were tagged with metadata denoting the conductances of interest (Cooper, Mirams, & Niederer, 2011), which results in auto-generated methods for changing the channel conductances in the resulting C++ code. The equations were solved using the adaptive time-stepping CVODE solver (Hindmarsh et al., 2005), with relative and absolute tolerances of 10^–6^ and 10^–8^ respectively, and a maximum time step of less than the stimulus duration. Adaptive time-stepping solvers offer significant speed and accuracy improvements over ‘traditional’ fixed time step solvers for numerically stiff systems such as cardiac action potential models. The software is a custom-made program based on the open-source Chaste library (Mirams et al., 2013) and its ApPredict (action potential prediction) module.

For the interested reader we have made the following resources available: the IC_50_ datasets, the action potential simulation software; and the scripts for generating the figures presented in this article. These can be downloaded as a ‘bolt-on project’ for Chaste (written to work with version 3.2) from http://www.cs.ox.ac.uk/chaste/download. Further instructions on downloading and using the code can be found in Supplementary Material S1.3.

### 2.5 Thorough QT study results

Calculated free plasma concentrations during the TQT study are given in a separate spreadsheet (Supplementary Material S2), based on data gathered for the Gintant (2011) study. The spreadsheet implements the necessary calculations for calculating molar free plasma estimates from maximum plasma concentration (‘C_max_’), percent plasma binding, and molecular weight. The equations used for calculations are given in Supplementary Material S1.4. The change in QT that was used for comparison with simulation predictions is the mean change in QTc, at the highest dose tested in the TQT study, as reported in Gintant (2011).

## 3 Results

In this section we present the results of the ion channel screening, followed by the simulations based upon those screens, and then analyse their predictions of TQT results.

### 3.1 Ion channel screening

Table 1 shows the pIC_50_ values (–log_10_ of IC_50_ values in Molar) fitted to the concentration effect points from each ion channel screen. We also display the manual hERG patch clamp values taken from Gintant (2011), which were collated from regulatory submission document GLP studies (ICH, 2005). Note that an IC_50_ > 10^6^ μM (or equivalently pIC_50_ < 0) would indicate a very weak (or no) compound effect on an ion current. When this was the case, we have ‘rounded’ and we show this in Table 1 as pIC_50_ = 0 for clarity. N.B. using pIC_50_ = 0 corresponds to just 0.01% channel block at our top concentration of 100 μM, and so these values were also used in the simulations, even when we suspect no activity at the channel.

When we compare the independent screens shown in Table 1, certain screens are very consistent (e.g. pIC_50_ of 6.0, 5.9 and 5.9 for hERG with Paliperidone), whilst others show wide variation (e.g. 5.0 and 0.0 for KCNQ1 with Duloxetine). Further screening of this type using a wider variety of assays would be valuable to establish the most reliable platforms.

### 3.2 Action potential simulation results

Fig. 3, Fig. 4 show a summary of the action potential prolongation results for a subset of the compounds, based upon the three different datasets. These compounds were selected to indicate representative cases where the simulations underestimate the TQT study results (Fig. 3), and cases where the predictions are more accurate (Fig. 4). Results for all of the individual compounds are shown in Supplementary Material S1.1.

In Fig. 3 we see the results for Alfuzosin and Lapatinib. The lines and shaded regions denote the three different model predictions, and the red circle (highlighted with black dashed lines) is the TQT result. In the case of Alfuzosin the models are not predicting any change in APD_90_ at the estimated TQT concentration (< 10^–2^ μM), but a correct prolongation is predicted at much higher concentrations. For this compound, the predictions are similar with all three datasets, with possibly the Barracuda set closest to TQT. Fig. 3 also shows results for Lapatinib. The Q and B&Q2 results similarly underestimate block, but in this case using manual patch hERG IC_50_ values significantly improves predictions, due to a stronger hERG block (see Table 1).

In Fig. 4 we show two further examples, where simulation predictions are more accurate. For Maraviroc the prediction is accurate for all data sources, with a very small prolongation observed at the TQT concentration. Sitagliptin is an example of prolongation being predicted with reasonable accuracy by all the datasets, again the M&Q dataset providing the closest fit to TQT results.

The different models sometimes provide different predictions. This is consistent with our observations of their single-channel block behaviour shown in Fig. 2. The 95% credible regions become wide when there is ‘overlap’ in the probability distribution of different ion channel pIC_50_ values, due to assay variability: for instance, hERG block could become significant before, at the same time, or after CaV1.2 block. At the same time, the different models are more/less sensitive to the different ion channel blocks, and so a wide uncertainty based on assay variability is also associated with divergence in model predictions. The Grandi et al. (2010) model appears more likely to predict shortening than the other two models, as one might expect by examining Fig. 2, since it is relatively insensitive to I_Kr_ and I_Ks_ block, and highly sensitive to I_CaL_ block.

To separate these effects, and select models that are most reliable for drug studies, will therefore require data with low variability.

#### 3.2.1 Contingency tables

In Table 2 we use the O'Hara et al. (2011) model predictions, at the estimated TQT concentration, and examine whether or not 5 ms prolongation is observed in TQT vs. simulation. For clarity, not all results are shown here in the main text; the full set of contingency tables can be found in Supplementary Material S1.5. Results shown in Table 2 for comparison of whether or not we achieve prolongation > 5 ms at the expected concentration using Quattro data are poor: there is a very low sensitivity of 14%.

**Table 2:**
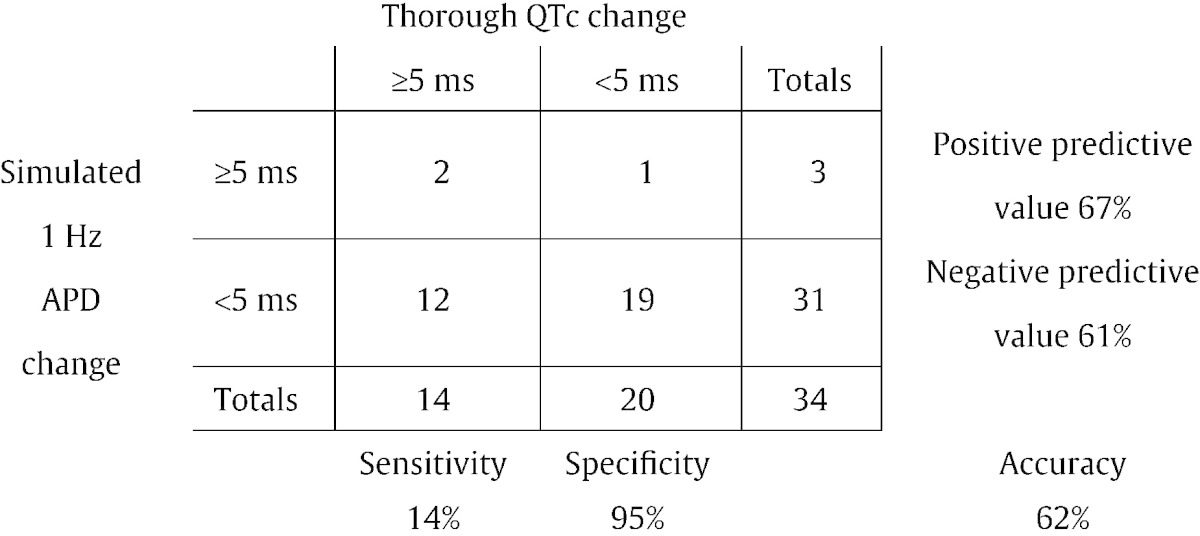
A contingency table for O'Hara et al. (2011) model predictions based on the IonWorks Quattro dataset. A match is defined as agreement at the estimated TQT concentration.

Examining the action potential vs. concentration curves for each compound (see Fig. 3, Fig. 4 and the full results in Supplementary Material S1.1) suggests that low sensitivity is not due to models being unable to predict prolongation, but rather to simulation predictions underestimating the APD prolongation at the estimated TQT concentration.

To test this, we allowed ‘success’ to take a more relaxed definition: of ‘agreement within a fold-change’ in the estimated concentration. One could interpret this as drawing ‘error bars’ around the TQT concentrations, and accepting model predictions falling within these. Table 3 presents a second contingency table as an example, looking for agreement within a 100-fold change in estimated TQT concentration. Increasing the allowable concentration range can (by definition) only improve the performance, but we do observe a significant increase in the sensitivity for detection of 5 ms prolongation in TQT (and specificity of 100% in this case).

**Table 3:**
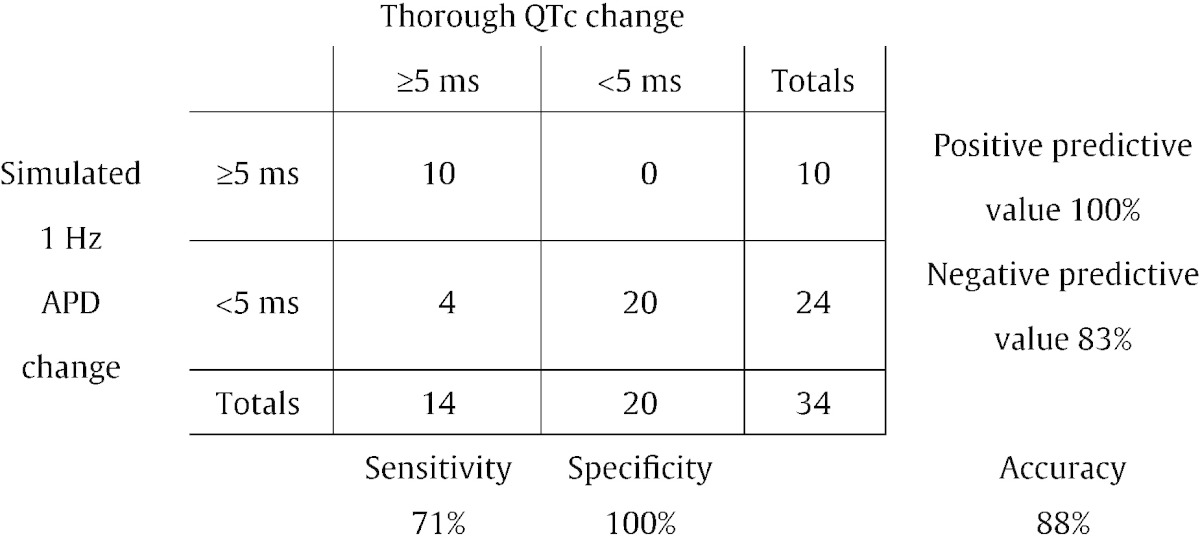
A contingency table for O'Hara et al. (2011) model predictions based on the IonWorks Quattro dataset. A match is defined as agreement within 100-fold of the estimated TQT concentration.

#### 3.2.2 Summary results

In Table 4 we summarise the sensitivity and accuracy of the models for different ranges of the ‘allowable concentrations’, and we also compare the effect of using the gold-standard manual patch clamp for hERG activity. As suggested by the Lapatinib example in Fig. 3, there is a trend for improved model predictions when using the manual hERG data.

**Table 4:**
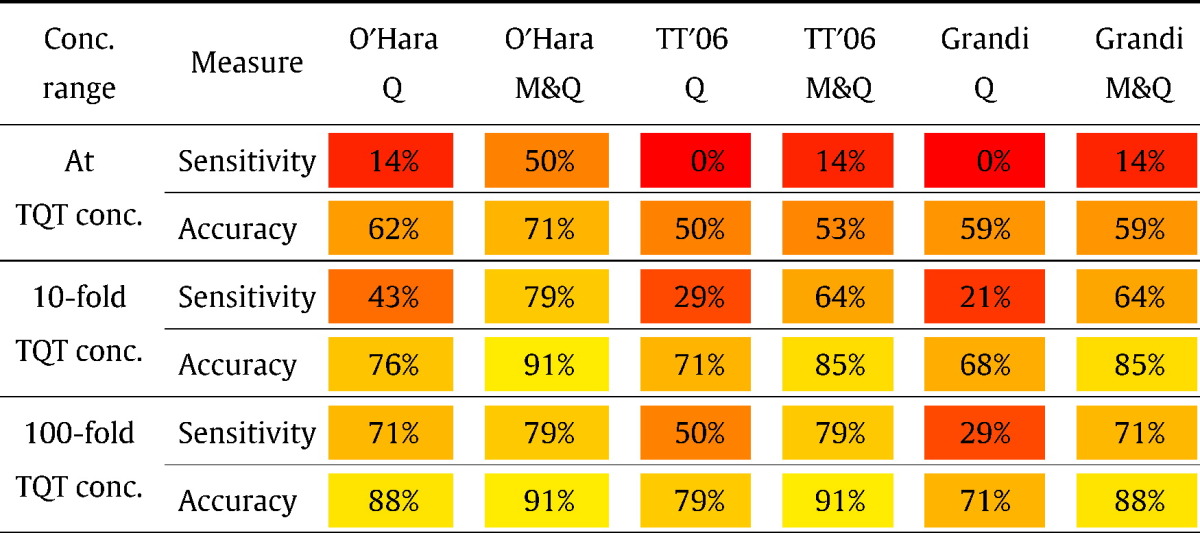
A summary of the models' performance with different datasets and ‘concentration windows’, when aiming to predict whether 5 ms prolongation is observed. Q — Quattro dataset, M&Q — Quattro with manual hERG (see Table 1).

For all models, predictions substantially improve both when considering a wider concentration range, and when using the M&Q dataset with GLP hERG IC_50_s. The worst performance is seen with the ten Tusscher and Panfilov (2006) and Grandi et al. (2010) models, for the Quattro data, when considering no range on the TQT concentration. The best performance is seen with the O'Hara et al. (2011) at 10-fold and 100-fold concentration windows and ten Tusscher and Panfilov (2006) model at the 100-fold concentration window, both when using the manual hERG dataset. In these cases we observe 79% sensitivity and 91% accuracy; we also obtain 100% specificity (see full contingency tables in Supplementary Material S1.5). This performance is an improvement on that found in Gintant (2011), based solely on hERG liability (using the same manual patch data), where the best marker was around 64% sensitive and 88% specific.

## 4 Discussion

In this study we have used ion channel screening data to simulate changes to action potential duration, and compared this with results of the human Thorough QT (TQT) study. At the estimated concentrations in the TQT study, simulations did not predict the same degree of prolongation as was observed in the study.

There are three leading possibilities for the observation that the simulations are underestimating TQT prolongation:

1. The concentrations estimated for the TQT study are underestimates.
2. Ion channel screens are underestimating compound-induced ion current reduction.
3. The mathematical models are underestimating changes in APD.

Below we discuss a number of reasons for why we believe these are ranked in order of likelihood.

Firstly, we undertook a similar study using IonWorks Quattro data and predicting changes to rabbit wedge QT using similar techniques and models (Beattie et al., 2013). In the ex-vivo rabbit wedge study, the concentrations of the compounds being perfused into the wedge tissue are known fairly accurately. In that study we observed sensitivity and specificity in the 70–80% ranges, in line with that observed when increasing the ‘concentration window’ in this study.

Secondly, our results show that using the manual patch clamp results from GLP regulatory submission documents substantially improves our predictions. Gillie, Novick, Donovan, Payne, and Townsend (2013) evaluated the IonWorks Barracuda screen for detection of hERG block; whilst block was consistently detected, this modern screening machine can report IC_50_s up to two orders of magnitude larger than manual patch results (see Gillie et al., 2013, Figure 8).

On the third point, the Beattie et al. (2013) study consistently estimated the concentration at which 10% prolongation of rabbit wedge QT would occur (to around half an order of magnitude, see Figure 2 of that paper). This suggests that the mathematical models are capable of predicting small changes in prolongation of repolarisation with some accuracy, when given similar data and evaluated against well-known concentrations.

The different models provide different predictions, consistent with what one may have predicted by looking at Fig. 2. The hERG pIC_50_ is often the strongest affinity in the screening panel (Table 1). Together with the O'Hara model's sensitivity to hERG block (Fig. 2), this means that prolongation tends to be predicted at lower concentrations using O'Hara than with the other models. In the case of multi-channel effects, the Grandi model (which shows little prolongation under I_Kr_ and I_Ks_ block) tends to show shortening more readily in the presence of any I_CaL_ blocking.

We tended to observe slightly better results with the O'Hara et al. (2011) model, but whether this is an accurate representation of its increased ability to predict drug effects is unclear: the model could be performing well by overestimating block effects at underestimated concentrations. The best results we found were with the O'Hara et al. (2011) model, using manual hERG data, within a 10-fold concentration window. Differences in the methods and data used for calibrating maximum ion channel conductance values during the original action potential model construction are likely to be the primary cause of different predictions here, with different ion channel formulations also playing a role. Further work is needed to refine the techniques used for model calibration, perhaps including the gathering of further novel human-based data.

### 4.1 Limitations/challenges

As discussed above, comparison of simulations with rabbit wedge QT results (Beattie et al., 2013) using the same type of screening data were more successful — perhaps because concentrations were known more accurately in that preparation. Some human ex-vivo ventricular wedge experiments, applying compounds at more accurately known concentrations, would be valuable to clarify this.

In terms of using a cellular rather than tissue simulation, here we directly compared the absolute prolongation of APD_90_ with the absolute change in QT interval. As part of the Beattie et al. (2013) study, we performed a simulation study of one-dimensional pseudo-ECG QT change and compared this with APD_90_ change. The results suggested an excellent correspondence between APD and QT changes, and that a ratio of ΔAPD_90_:ΔQT of 1:1.35 provides the line of best fit.^2^ This suggests that a simple rescaling of APD_90_ to improve prediction of QT may be in order for future refinement.

Note that the concentration used was assumed to be the free molar concentration corresponding to the C_max_ value. Using this concentration ignores the timing of QT measurements, active metabolites, and any effects leading to compound accumulation in cardiac tissue, but these data were not readily available.

There are many possible compound effects that were not being screened for, and hence could not be picked up in in-silico predictions, no matter how accurate the models. An example would be changes in ion channel trafficking to the membrane, which are not screened for as standard. Certain compounds may have known additional affects that could explain inaccurate predictions: in the case of Alfuzosin (Fig. 3) TQT prolongation may be caused by sodium channel activation (Lacerda et al., 2008). This could be screened for, but isn't something we have included here.

Of the 34 drugs studied, only three (Darifenacin, Desvenlafaxine, Etravirine) had simulated predictions of prolongation instead of shortening (of 2–7 ms) for all models and datasets. There were no compounds for which simulations predicted shortening instead of prolongation across all combinations. This proportion of 3/34 gives an impression of the background rate of confounding compounds, in which simulated predictions are highly inaccurate. These are probably down to factors such as additional channel blocks, interaction with nervous system etc. which make the simulated compound effects an incomplete representation of the compounds' true actions. The true proportion of drugs with off-target effects that we could not capture could be lower, as predictions here may be inaccurate simply due to underestimated channel potencies.

Because screening will always target a subset of components, later experimental safety tests will remain crucial to detect off-target and more subtle compound-induced effects. Stem-cell derived cardiomyocytes are one possibility for largely replacing the role of animal models for later-stage safety tests in the future. We agree with the comment in Kleiman, Shah, and Morganroth (2014), that “[computer models]… need to be standardized, regulated and widely available before they are adopted to support sponsor and regulatory decisions”.

### 4.2 Choosing ion channels to screen

It is sensible to ask “which ion channels should we screen”? We consider important factors in the answer to this in the sections below.

#### 4.2.1 How much impact can each ion channel have?

For our output of interest, how much can block of a particular channel influence the predictions? In this case, we are interested in predicting APD changes, it is evident from Fig. 2 that (depending on the model choice) I_Kr_, I_CaL_ and perhaps I_Ks_ block could have large effects on APD.

At the degree of block likely to be encountered, block of (solely) I_Na_ and I_to_ have much less impact than those of the other channels, and so a choice could be made not to screen these. But more mechanistic predictions of pro-arrhythmic risk, other than simply APD prolongation, may be sensitive to the apparently-small changes we observed. Indeed, sodium channel blockers have been seen to prolong the QRS complex, potentially leading to increased pro-arrhythmic risk via conduction slowing or block, rather than delayed repolarisation (Gintant, Gallacher, & Pugsley, 2011). It is also worth noting that APD is not a linear function of channel block — blockade of I_Na_ and I_to_ could have large effects when another channel is also being blocked. A more ‘global’ evaluation of the simulation output's sensitivity to each channel block (under the influence of different combinations of block on the other channels) would be needed before concluding a channel cannot significantly influence the outcome of interest.

In contrast, additional ion channels — such as IK_1_ — can have a large effect on the action potential (Fig. 2). But these channels may not be blocked by a large enough proportion of compounds to consider screening them as standard, as discussed below.

#### 4.2.2 How likely is each channel to be blocked?

Some ion channels, pumps and exchangers are historically blocked by very few compounds. The outcome of ‘missing an effect’ in these rare cases is likely to be no more severe than progressing such a compound to later, more expensive, safety testing, and picking up the effect there. The economic cost of screening for additional effects on such ion currents may therefore outweigh the cost of missing an ion current effect.

There is also the variability, sensitivity and specificity of such screens to consider. In the case of an ion channel being blocked by as few as 1 in 10,000 compounds, the chance of a positive screening result being a ‘false positive’ is likely to far outweigh the chance of it being a ‘true positive’. A cost benefit analysis could be performed for each ion channel screening assay, based on: its variability, sensitivity and specificity; historical compound liability; and the cost of ‘missing’ an adverse interaction with this channel, and progressing to the next stage of testing.

### 4.3 Conclusions

The aim of our line of research, and the Comprehensive in-vitro Pro-arrhythmia Assay (CiPA, Sager et al., 2014), is to provide more human-relevant assessment of pro-arrhythmic risk as early as possible in drug development. Instead of using animal-based experimental models, more accurate predictions for human QT and pro-arrhythmic risk could be obtained by using human mathematical action potential simulations, based on data from human ion channel protein screens, in the near future. The performance of such simulations for cardiac safety assessment is going to be sensitive to both the choice of action potential model, and the choice of screening data.

There are layers of complexity that are ignored by simply screening four or five ion channels and predicting a human body surface response using these models. Yet the levels of success we observed here suggest that the majority of biophysical processes which are contributing to QT prolongation are captured by screening a handful of ion channels, and are integrated appropriately by the mathematical models. This is very encouraging for future refinement of this work, and extending the approach to examine pro-arrhythmic risk mechanistically.

## Appendix A Supplementary data

Supplementary material S1 full methods description and detailed results for individual compounds.

Supplementary material S2 a spreadsheet of references and calculations for TQT concentrations used in this study.

## Footnotes

1 These simulations were performed by the Oxford Cardiac Electrophysiology Web Lab (https://chaste.cs.ox.ac.uk/FunctionalCuration), an open online resource for exploring the behaviour of a range of action potential models, under a range of simulated experimental protocols. Users can also upload a new model and subject it to the existing protocols, or examine the response of the existing models to a new protocol.

2 Results are featured on a short blog article at http://mirams.wordpress.com/2014/03/21/apd_vs_qt/.
