# Supplementary material for: Prediction of Thorough QT study results using action potential simulations based on ion channel screens
Source: J Pharmacol Toxicol Methods. 2014 Nov;70(3):246–54. doi: 10.1016/j.vascn.2014.07.002 (PMC4266452; doi:10.1016/j.vascn.2014.07.002)
Supplement: Supplementary material S1 — full methods description and detailed results for individual compounds. [file mmc1.pdf]

Supplementary Material S1 for: Prediction of Thorough QT study results using action potential simulations based on ion channel screens

Gary R. Mirams *et al.*

June 18, 2014

Contents

S1 Supplementary Material **1**
S1.1 Full APD results . . . 1
S1.2 Screening methods . . . 12
S1.2.1 AstraZeneca IonWorks Quattro screens . . . 12
S1.2.2 GSK Screening Methods . . . 15
S1.3 Open source simulation code . . . 19
S1.4 TQT study concentration calculations . . . 20
S1.5 Full contingency table results . . . 20

S1 Supplementary Material

S1.1 Full APD results

The result for four compounds are shown in Figures 3 and 4 of the main text. Figures S1–S30 are shown here, to give full results for all the remaining compounds.

In each plot the colour key is the same for the action potential models: blue — O'Hara; red — ten Tusscher; green — Grandi. Estimated 95% credible regions are shown around each line which capture uncertainty due to screening assay variability. The clinical trial results are shown with red circle, highlighted with a black dashed horizontal line; the estimated free plasma concentration associated with this is shown with a vertical dashed black line. The 5ms 'cut-offs', used in contingency table calculations, is shown with a horizontal blue dotted line. Each of the three data source variants we used is plotted separately:

- Q — IonWorks Quattro screen for all four channels (see AZ methods in section S1.2.1).
- B & Q2 — IonWorks Barracuda screen for hERG and CaV1.2, Quattro for NaV1.5 and KCNQ1 (see GSK methods in section S1.2.2), note not all compounds were screened using these assays.
- M & Q — Manual patch for hERG, AZ IonWorks Quattro for the other channels.

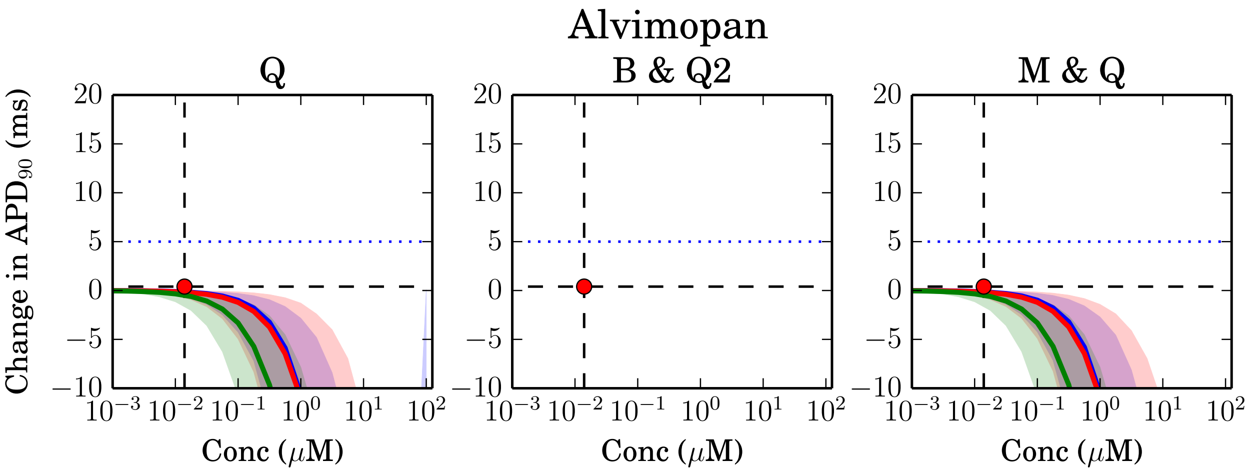

Figure S1: Simulated change in action potential duration vs. Alvimopan concentration.

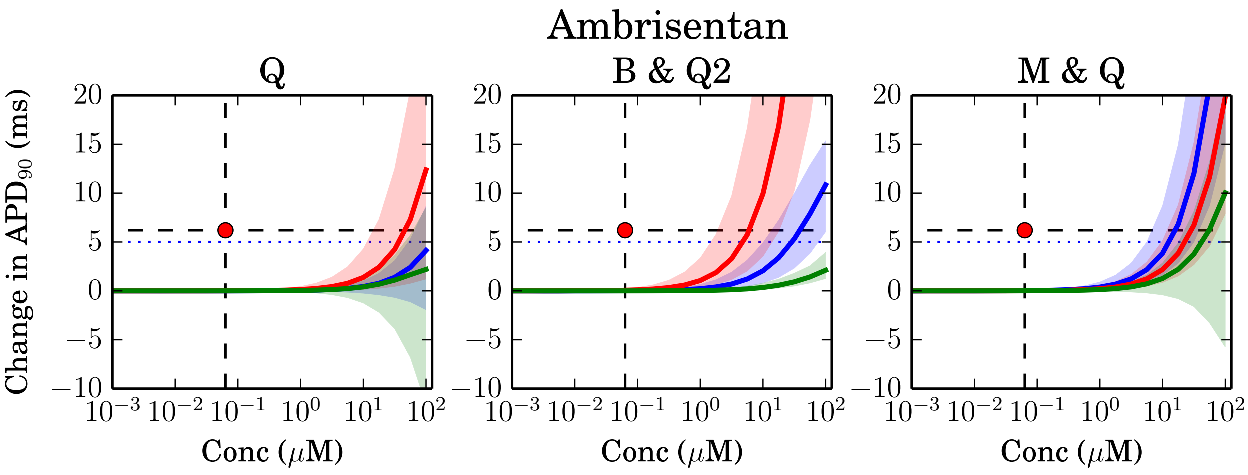

Figure S2: Simulated change in action potential duration vs. Ambrisentan concentration.

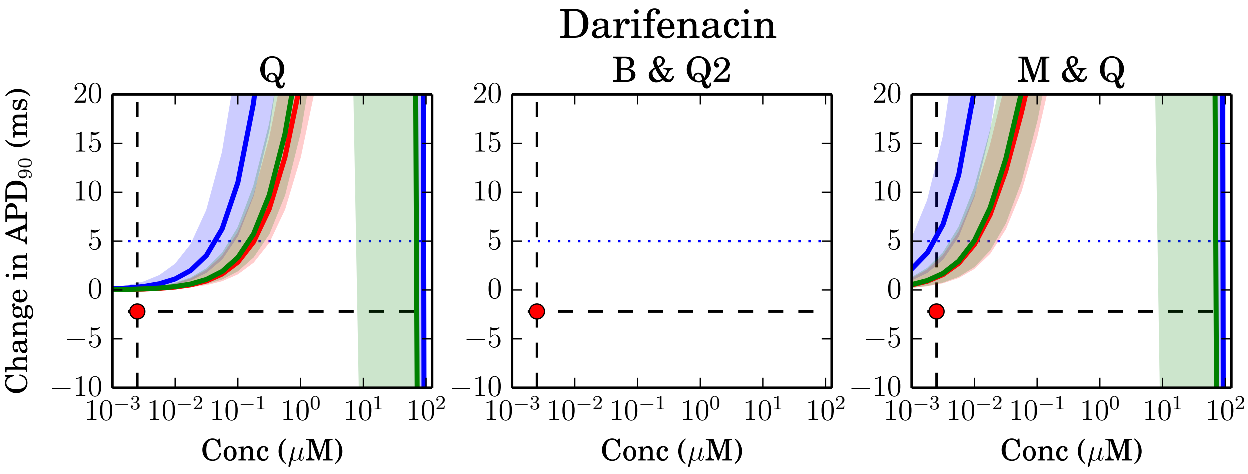

Figure S3: Simulated change in action potential duration vs. Darifenacin concentration.

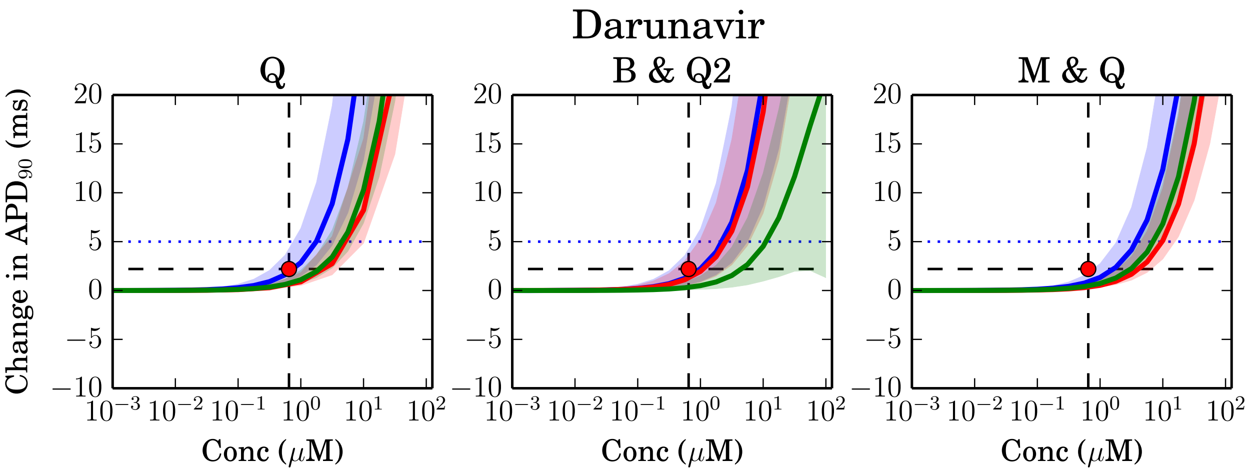

Figure S4: Simulated change in action potential duration vs. Darunavir concentration.

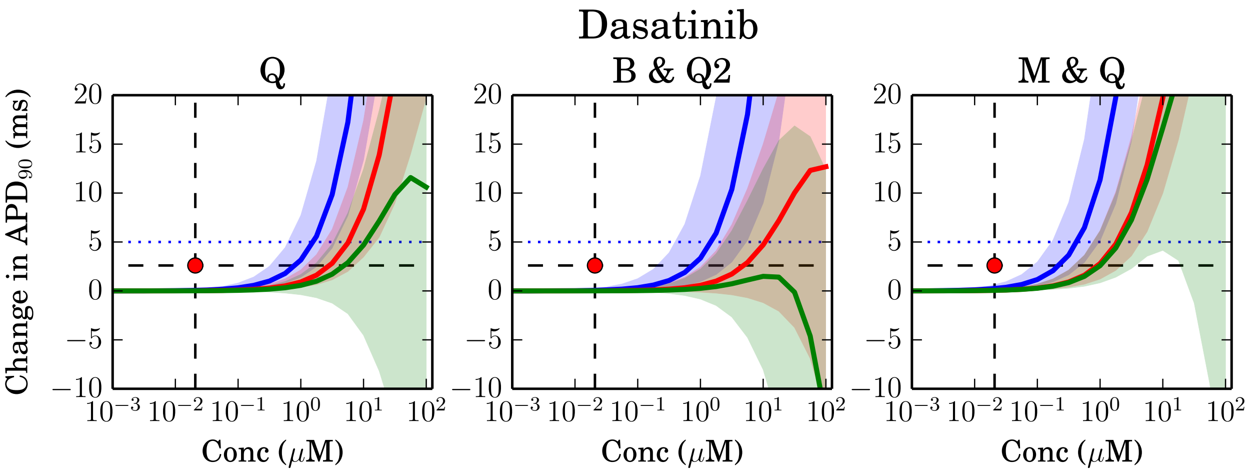

Figure S5: Simulated change in action potential duration vs. Dasatinib concentration.

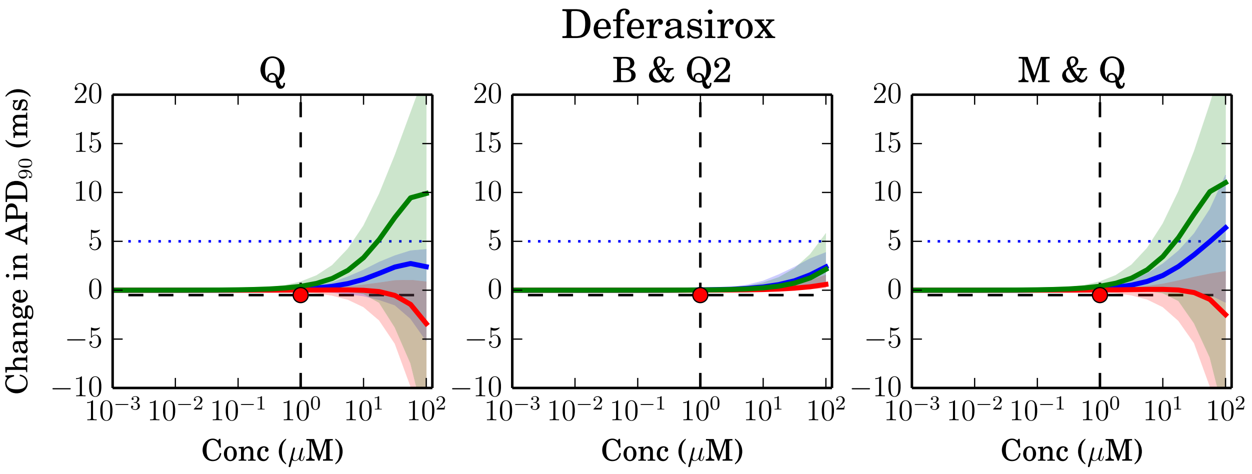

Figure S6: Simulated change in action potential duration vs. Deferasirox concentration.

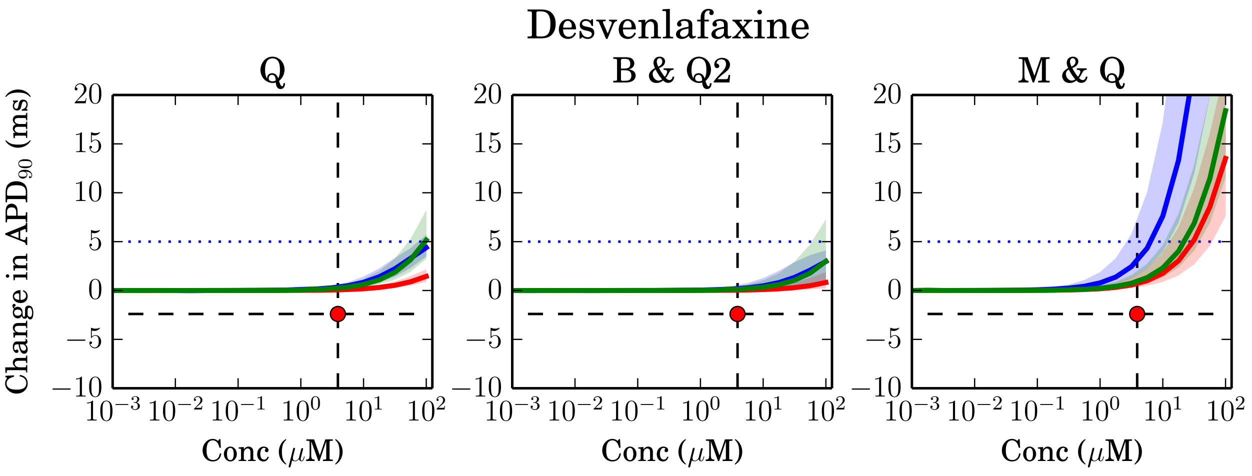

Figure S7: Simulated change in action potential duration vs. Desvenlafaxine concentration.

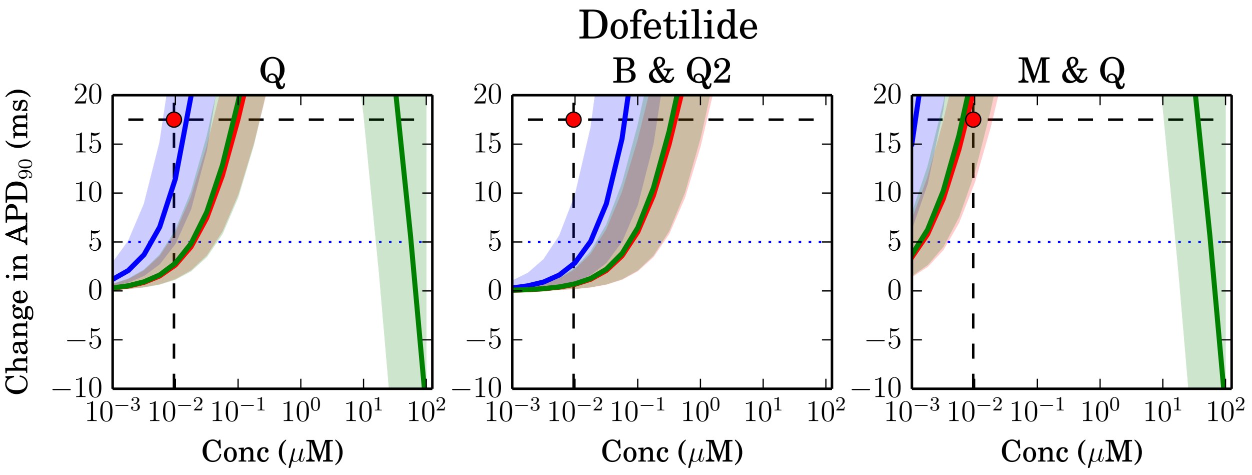

Figure S8: Simulated change in action potential duration vs. Dofetilide concentration.

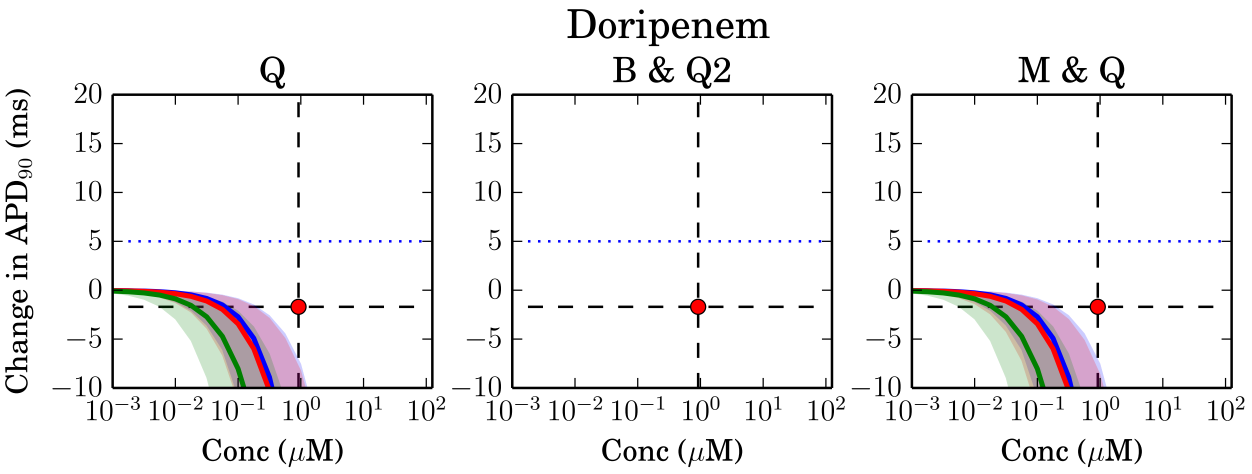

Figure S9: Simulated change in action potential duration vs. Doripenem concentration.

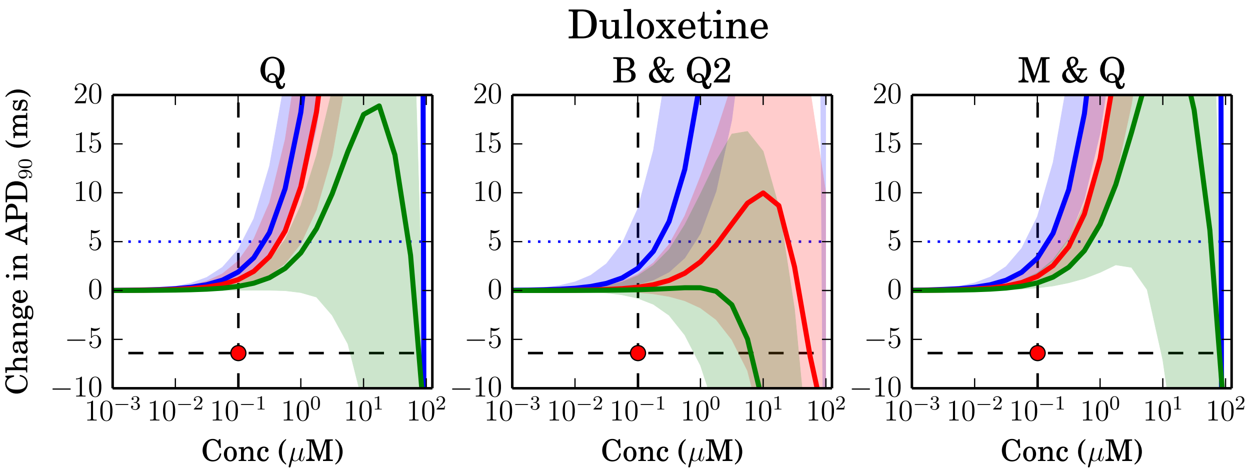

Figure S10: Simulated change in action potential duration vs. Duloxetine concentration.

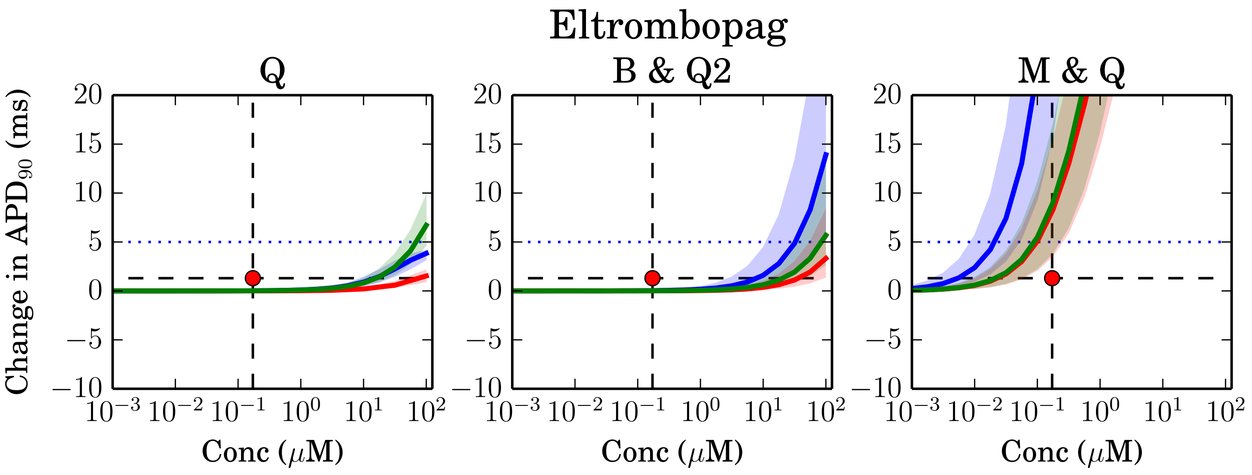

Figure S11: Simulated change in action potential duration vs. Eltrombopag concentration.

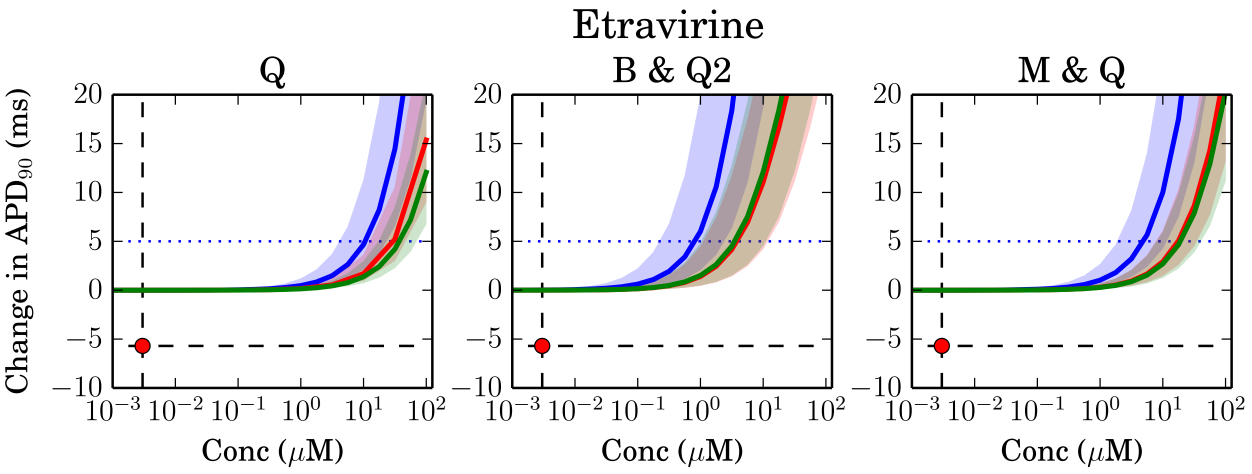

Figure S12: Simulated change in action potential duration vs. Etravirine concentration.

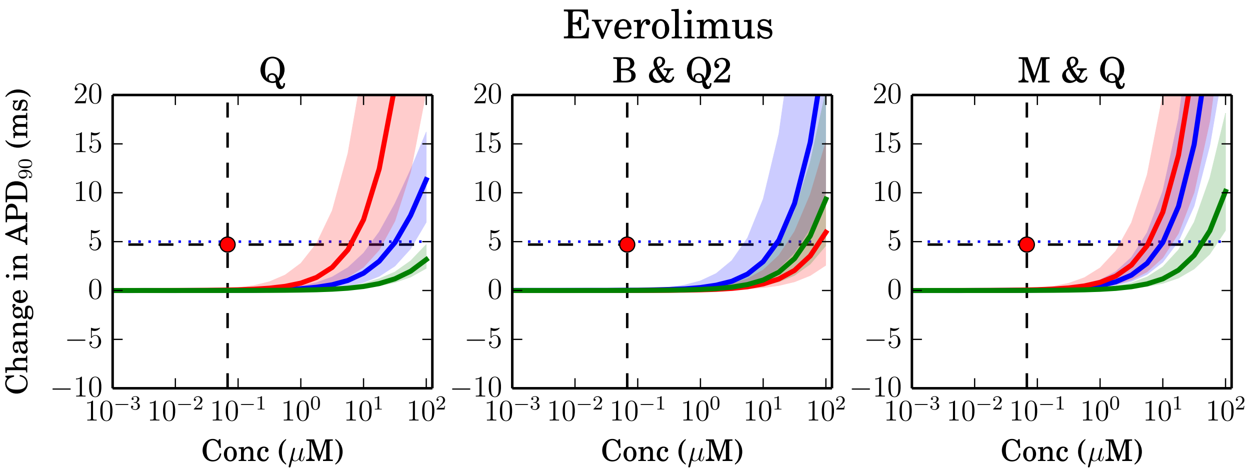

Figure S13: Simulated change in action potential duration vs. Everolimus concentration.

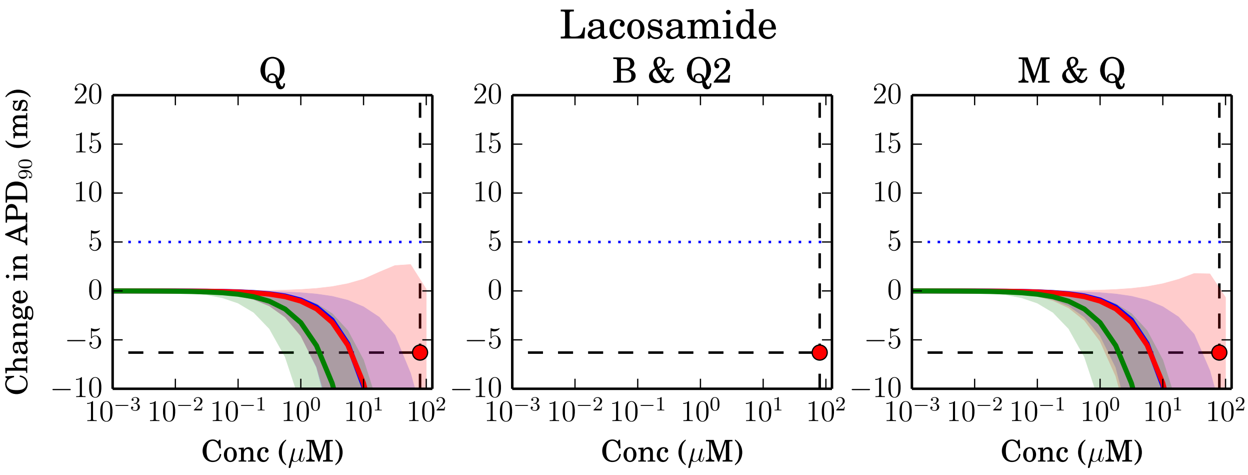

Figure S14: Simulated change in action potential duration vs. Lacosamide concentration.

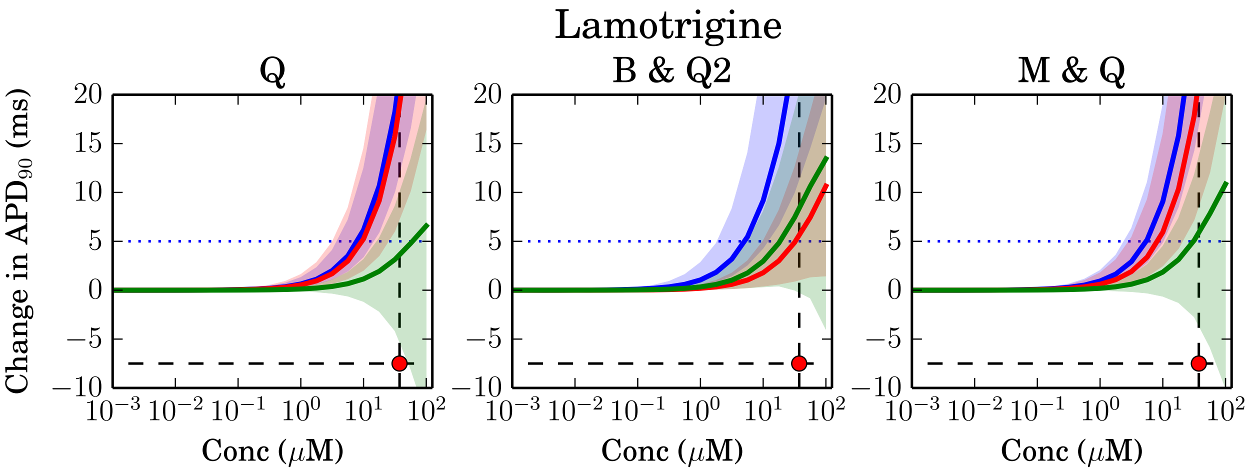

Figure S15: Simulated change in action potential duration vs. Lamotrigine concentration.

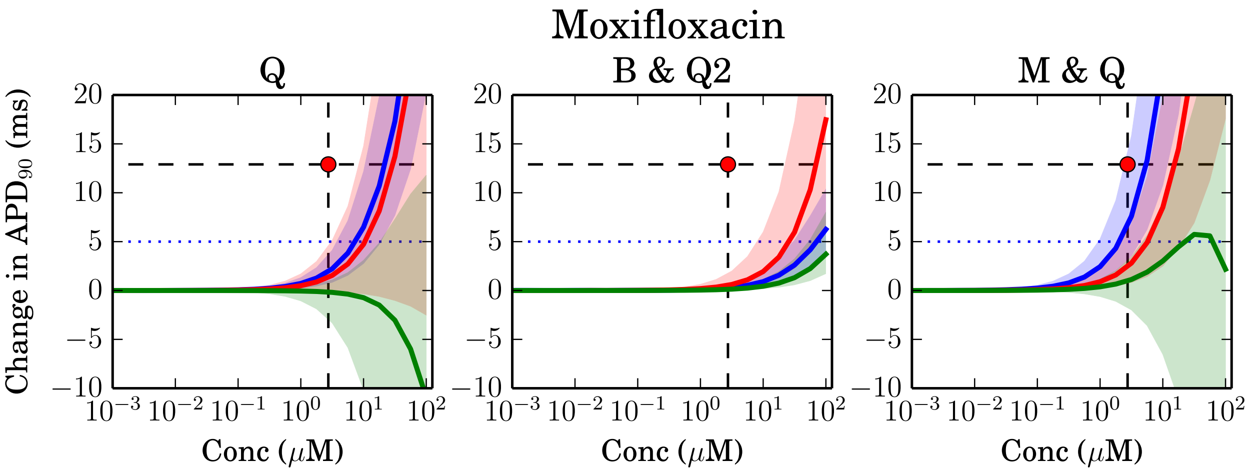

Figure S16: Simulated change in action potential duration vs. Moxifloxacin concentration.

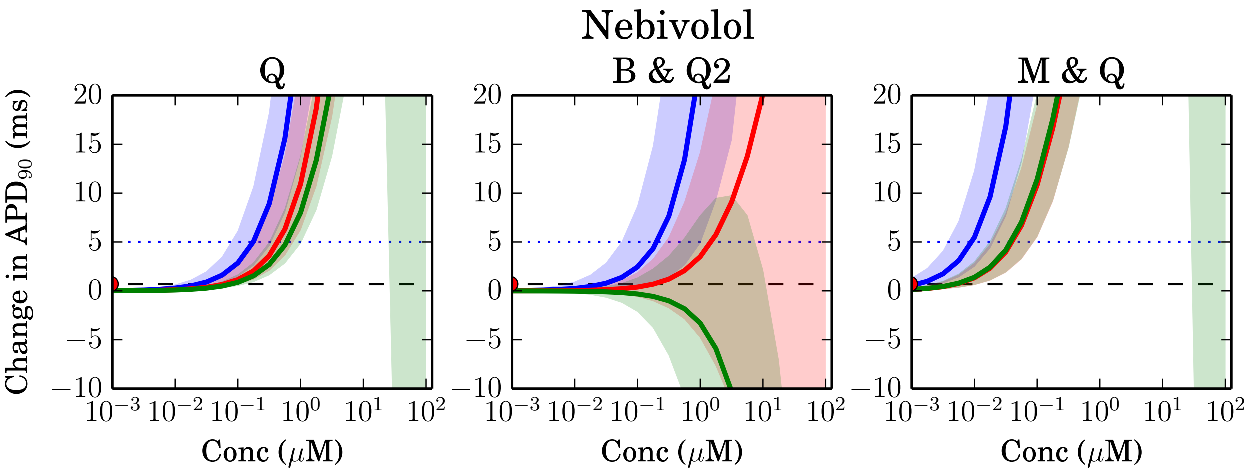

Figure S17: Simulated change in action potential duration vs. Nebivolol concentration.

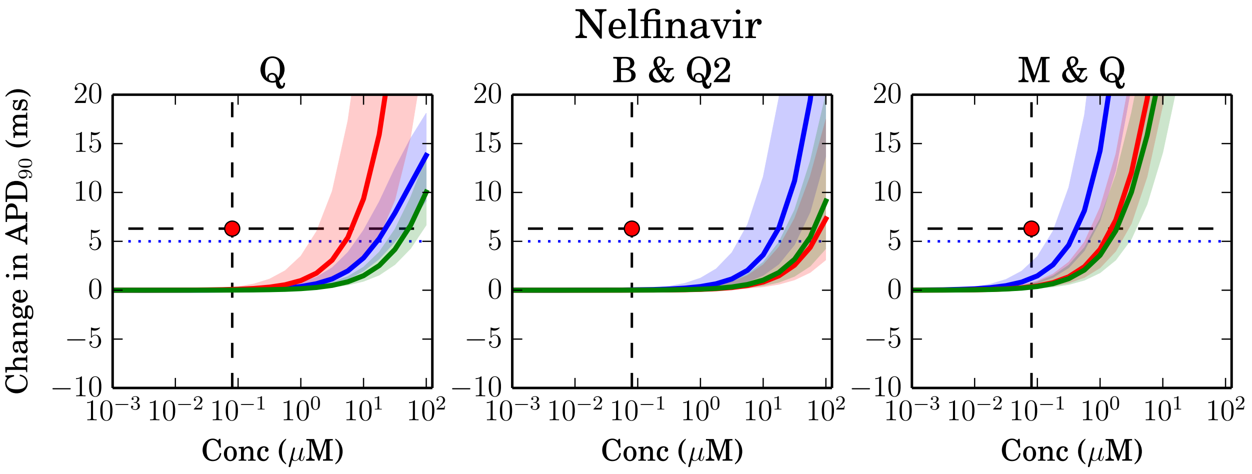

Figure S18: Simulated change in action potential duration vs. Nelfinavir concentration.

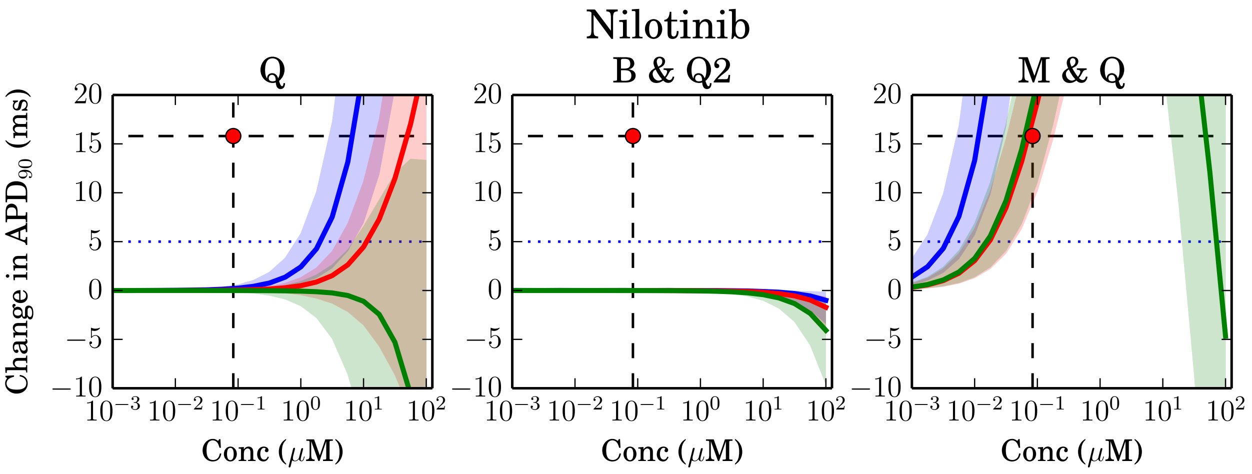

Figure S19: Simulated change in action potential duration vs. Nilotinib concentration.

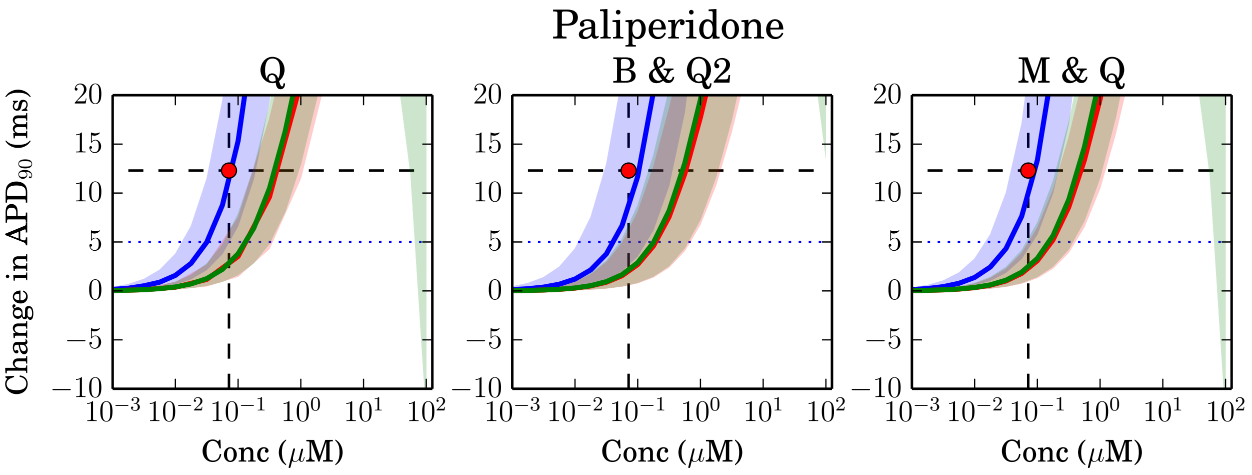

Figure S20: Simulated change in action potential duration vs. Paliperidone concentration.

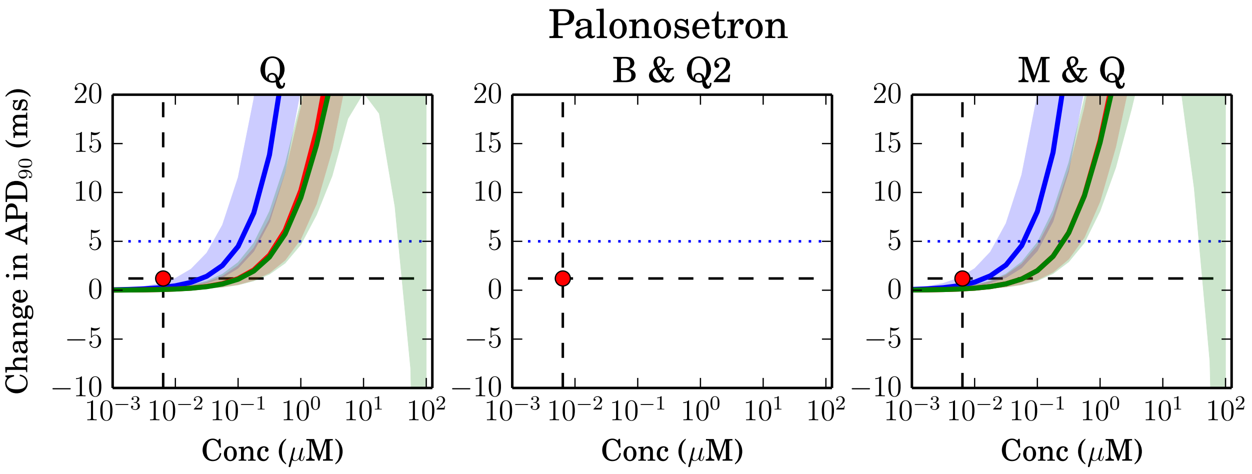

Figure S21: Simulated change in action potential duration vs. Palonosetron concentration.

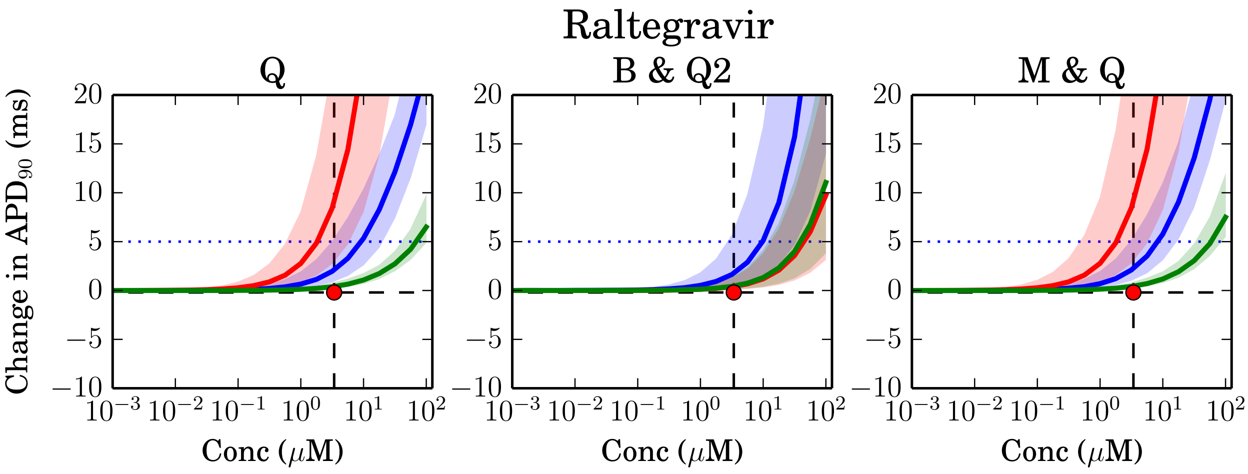

Figure S22: Simulated change in action potential duration vs. Raltegravir concentration.

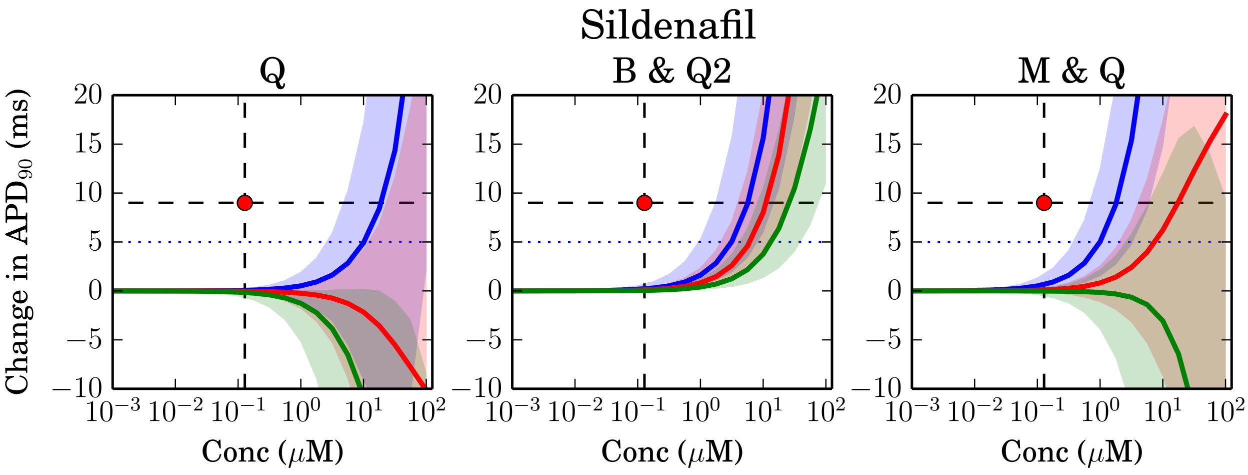

Figure S23: Simulated change in action potential duration vs. Sildenafil concentration.

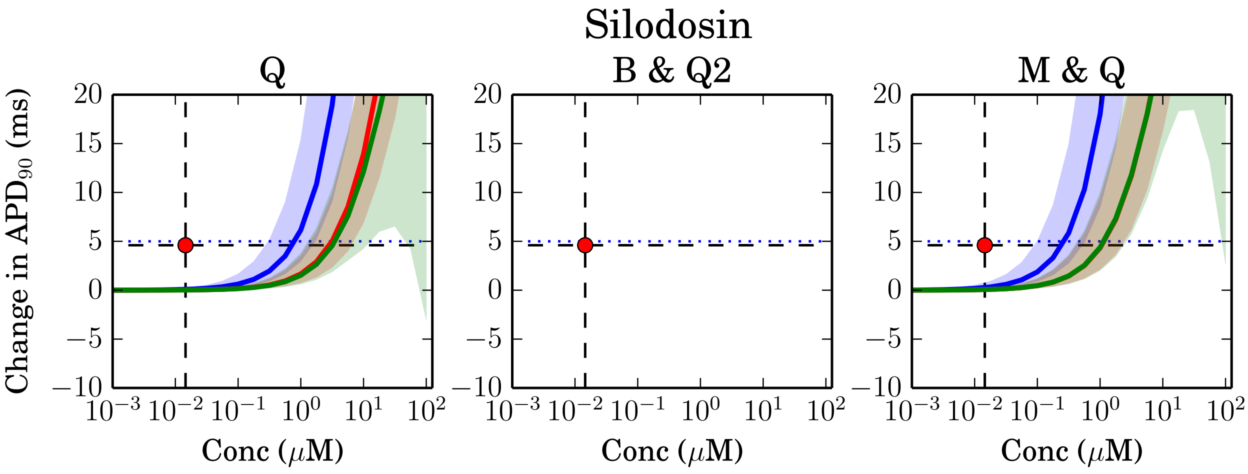

Figure S24: Simulated change in action potential duration vs. Silodosin concentration.

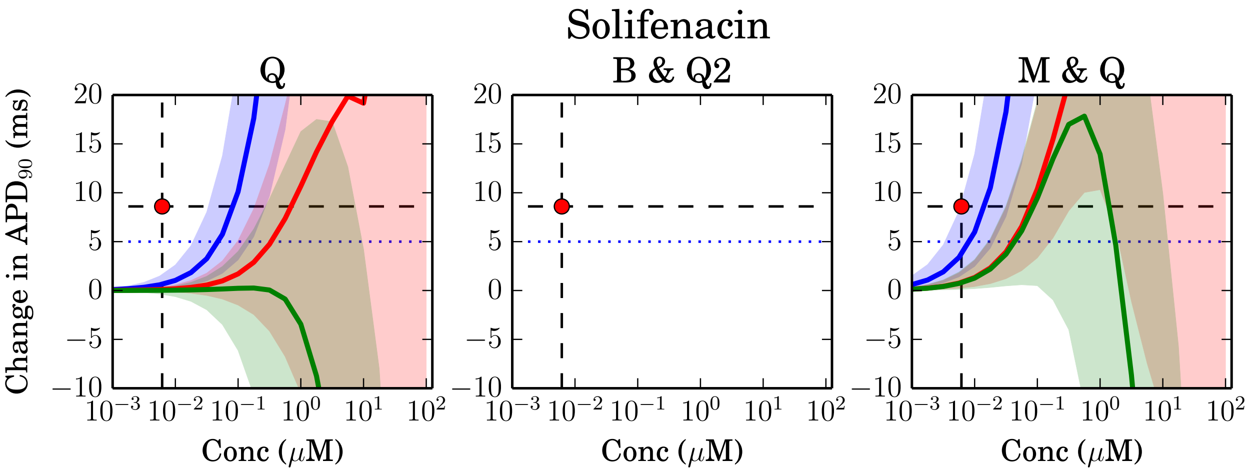

Figure S25: Simulated change in action potential duration vs. Solifenacin concentration.

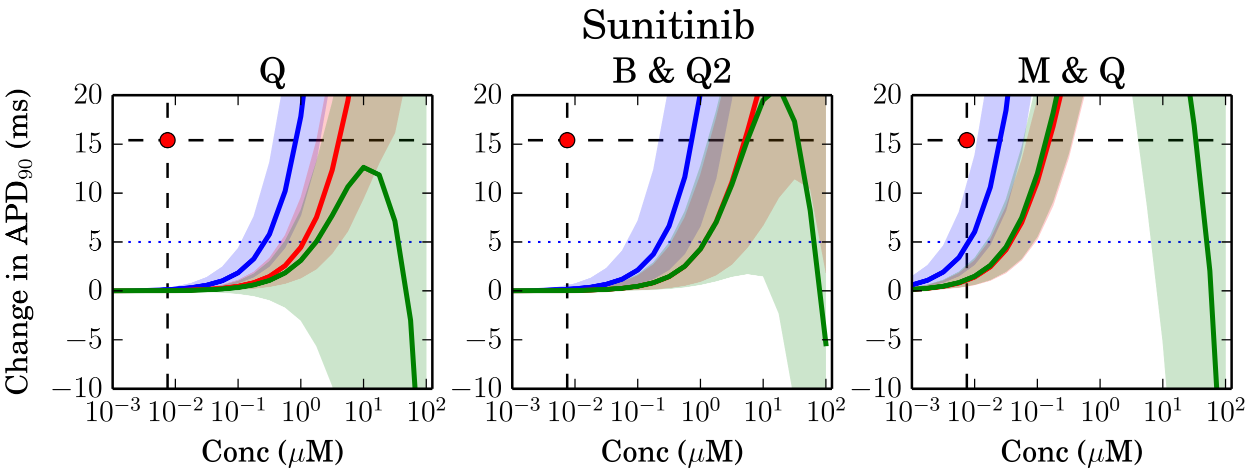

Figure S26: Simulated change in action potential duration vs. Sunitinib concentration.

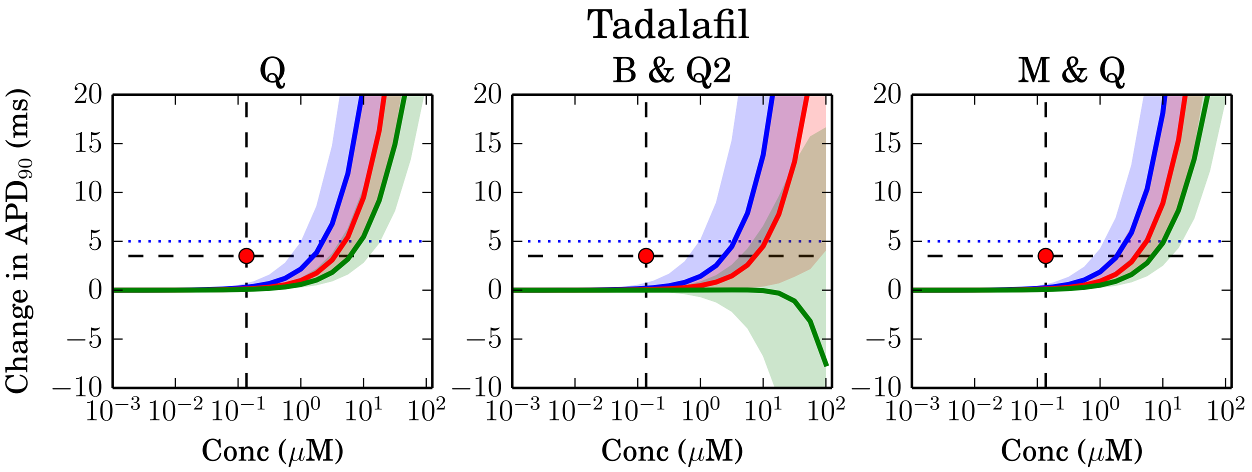

Figure S27: Simulated change in action potential duration vs. Tadalafil concentration.

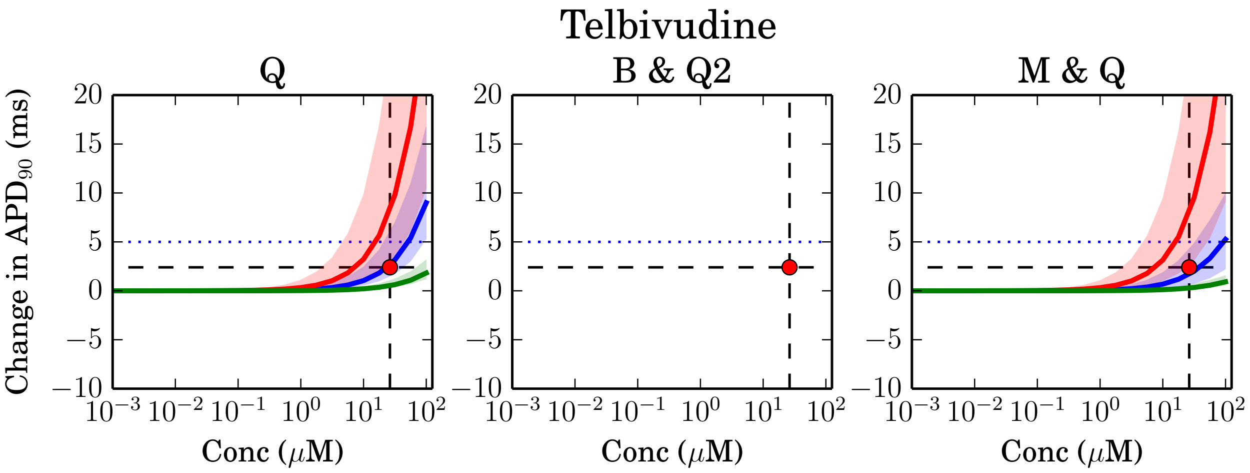

Figure S28: Simulated change in action potential duration vs. Telbivudine concentration.

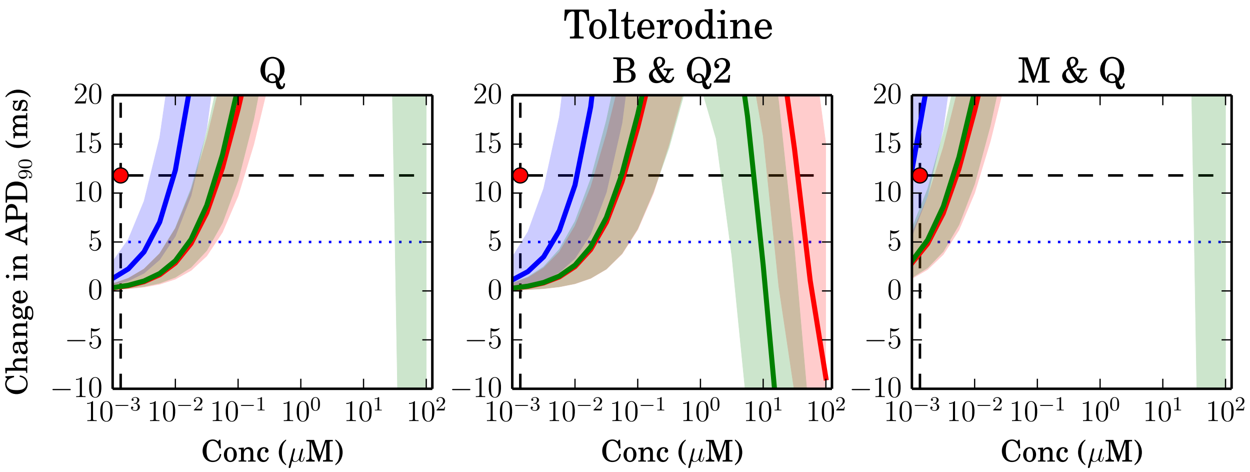

Figure S29: Simulated change in action potential duration vs. Tolterodine concentration.

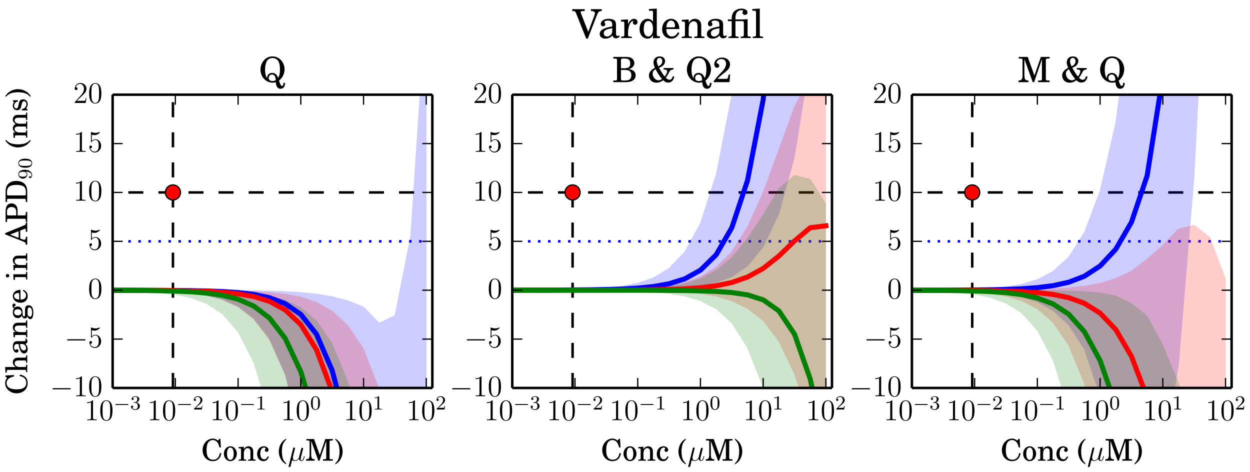

Figure S30: Simulated change in action potential duration vs. Vardenafil concentration.

S1.2 Screening methods

Section 2.1 of the main text refers to two ion channel screening datasets:

- AstraZeneca IonWorks Quattro (34 compounds) — screening methods for the collection of this dataset were described in Elkins et al. (2013), they are reproduced below in section S1.2.1 for completeness.
- GlaxoSmithKline IonWorks Barracuda & Quattro (29 compounds) — screening was performed as detailed below in section S1.2.2.

S1.2.1 AstraZeneca IonWorks Quattro screens

These details in this section are as in Davies et al. (2012, Table 1), as the screens were undertaken by the same team at AstraZeneca, using the same procedure as that study.

Cell culture

Cells expressing hKv11.1 (hI_{ERG}):
CHO K1 cells[1] were stably transfected with human ERG cloned into the pcDNA3.1 vector. They were grown in Hams F-12 nutrient mixture and L-glutamine supplemented with 10% FCS and 600μg/ml Hygromycin. Cells used in the IonWorks were incubated at 37℃ for 24 h and then incubated at 30℃ for 48–72 h.

Cells expressing hNaV1.5 (hI_{Na}):
CHO K1 cells[1] were stably transfected with human Nav1.5 cloned into the pcDNA3.1 vector.. They were grown in Hams F-12 nutrient mixture and glutamax supplemented with 10% FCS and 1,000μg/ml geneticin. Cells used in the IonWorks were incubated at 37℃ for 48 h.

Cells expressing hKv4.3/hKChIP2.2 (hI_{to}):
CHO K1 cells[1] were stably transfected with human Kv4.3 cloned into the PGEN-IRES neo vector and human KChIP2.2 was cloned into the PGEN-IRES hygro vector. They were grown in Hams F-12 nutrient mixture and glutamax supplemented with 10% FCS, 1,100μg/ml geneticin, and 600 μg/ml hygromycin. Cells used in the IonWorks were incubated at 37℃ for 24 h, then at 30℃ for 48 h.

Cells expressing hKvLQT1/hminK (hI_{Ks}):
CHO cells purchased from Millipore (Cat no. CYL3007) were grown in Iscoves nutrient mixture and Glutamine supplemented with 10% FCS, 400μg/ml geneticin, 100μg/ml hygromycin, 2% HT supplement (50×), and 1% non-essential amino acids (100×). Cells used in the IonWorks were incubated at 37℃ for 24 h and then incubated at 30℃ for 24 h.

Cells expressing hCav1.2(hI_{Ca}):
CHO cells purchased from Chantest Corporation (Cat no CT6004) were grown in Hams F-12 nutrient mixture and L-glutamine supplemented with 10% FCS, blasticidin (10μg/ml), geneticin (G-418, 400μg/ml), hygromycin (250μg/ml), penicillin-streptomycin (100 units/ml; 100μg/ml), and zeocin (75μg/ml). Twenty-four hours before assay cells were induced with doxicycline to a final concentration of 1μg/ml and incubated for a further 4 hours at 37℃. They were then incubated at 30℃ overnight.

[1]Cells were generated by AZ Mölndal, as described by Persson et al. (2005a,b).

All cells were grown to semiconfluence at 37℃ in a humidified environment (5% CO_2).

Preparation of cells for IonWorks

Cells expressing hI_{ERG} and hI_{Na}[2]:
After the monolayer of cells was detached with Versene solution (∼3 ml, 1:5,000), cells were washed with PBS (Dulbecos phosphate containing Ca^{2+} /Mg^{2+}) and centrifuged at 50 g for 4 min. The supernatant was discarded and the remaining pellet of cells was re-suspended in of PBS. For hI_{ERG} (IonWorks) and hI_{Na} (Quattro) measurements, cell concentrations of 0.25×10^6 cells/ml and 1×10^6 cells/ml were used, respectively.

Cells expressing hI_{to}[2] and hI_{Ks}:
The method used was the same as that prescribed above, except for the following changes: cells were washed with PBS (no Ca^{2+}/Mg^{2+}) and incubated with 0.05% Trypsin/EDTA solution. Both cell lines were adjusted to 1×10^6 cells/ml (both run in Quattro mode).

Cells expressing hI_{CaL}:
After the monolayer of cells was washed with PBS, cells were detached with accutase and centrifuged at 1,100 g for 2 min. The supernatant was discarded and the remaining pellet of cells was re-suspended in HBPS containing 10 mM $BaCl_2$. (HBPS + Ba) to a concentration of 1.5 million cells/ml.

Measurements of currents

Cells are incubated for 3 minutes in the presence of a compound before acquiring the ion current data post-compound addition.

hI_{ERG}:
A holding potential of −70mV was applied for 20 s, followed by a 160 ms step to −60mV (allowing an estimated leak current to be measured), and a 100 ms step back to −70mV. The voltage was then stepped to +40mV for 1 s and a steady-state current was observed. A 2 s step down to −30mV, inducing the tail current, was then followed by a 0.5 s step to −70mV.

hI_{Ks}:
The voltage protocol consisted of a 5 s period holding at −80mV, a 100 ms step to −100mV (to measure an estimated leak current), a 100 ms step back to −80mV, followed by a 4 s step to +40mV, a 300 ms step to −40mV, and finally a 200 ms step to −80mV.

hI_{Na} and hI_{to}[3]:
The voltage protocol consisted of a 15 s period holding at −90 mV, a 160ms step to −100mV (to measure an estimated leak current), a 100 ms step back to −90mV, followed by 10 pulses each for a duration of 50 ms applied at 3 Hz. The first eight 50 ms pulses were to −20mV and the ninth and tenth pulses to +20 mV. 300 ms after the tenth pulse there was another longer pulse to +20 mV (1 s) with a final 300 ms step to −90 mV.

hI_{CaL}:

[2]When performing the CHO-hI_{Na}/CHO-hI_{to} duplex assay, the cell counts were determined and the cell concentration for both cell suspensions was adjusted to 1×10^6 cells/ml. The cells were mixed together to attain a 60:40 ratio hI_{Na}:hI_{to}. A single voltage pulse was applied to evoke the pre- and post-compound currents, and the degree of inhibition or stimulation was assessed by dividing the postscan current by the respective prescan current for each well.

[3]The degree of response for hI_{Na} current was assessed for both the first and eighth pulses, while the effect on hI_{to} was assessed for the eleventh pulse.

A holding potential of −65 mV was applied for 10 s, before depolarizing to 0 mV for 500 ms and a steady-state current observed.

Solutions

hI_{ERG}, hI_{Ks}, hI_{Na}, and hI_{to}:
The internal solution was composed of (in mM) 100 K-gluconate, 40 KCl, 3.2 $MgCl_2$, 3 EGTA , and 5 HEPES (pH 7.3 using 1 M KOH). The access solution was composed of (in mM) 140 KCl, 1 EGTA, 1 $MgCl_2$, and 20 HEPES (pH 7.3 using 1 M KOH), and 100 μg/ml of amphotericin B. PBS contains (in mM) 136.9 NaCl, 2.7 KCl, 8 Na_2HPO_4, 1.5 KH_2PO_4, 0.9 $CaCl_20.2H_2O$, and 0.5 $MgCl_20.6H_2O$.

hI_{CaL}:
Similarly to the other four currents, same internal solution was used. The access solution was composed of (in mM): KCl 140, EGTA 1, $MgCl_2$ 1 and HEPES 20 (pH 7.3 using 1 M KOH), 4 mM escin, 2 mM K_2ATP and 0.3 mM Na_2GTP. HBPS contains (in mM): 137 NaCl, 10 $BaCl_2$, 4 KCl, 10 HEPES, 10 Glucose 1 $MgCl_20.6H_2O$, pH 7.4.

Positive controls

hI_{ERG}:
Cisapride was solubilized in DMSO at a concentration of 3mM and further diluted in PBS to make a top test concentration of 10μM.

hI_{Na} and hI_{to}:
Flecainide was solubilized in DMSO at a concentration of 95mM and further diluted in PBS to make a top test concentration of 316μM.

hI_{Ks}:
XE991 was solubilized in DMSO at a concentration of 9.5mM and further diluted in PBS to make a top test concentration of 31.6μM.

hI_{CaL}:
Verapamil was solubilized in DMSO at a concentration of 30mM and further diluted in HPBS + Ba^{2+} to make a top test concentration of 100μM.

Serial dilutions

hI_{ERG}, hI_{Ks}, hI_{Na}, hI_{to}, and hI_{CaL}:
Each test compound was solubilized and serially diluted 7 times by half $\log_{10}$ units in DMSO as stock solutions. Each of these concentrations was then further diluted 100-fold in PBS (HBPS + Ba^{2+} for hI_{Ca}) in a 96-well plate. Each compound was then diluted threefold in PBS (HBPS + Ba^{2+} for hI_{Ca}) in the PatchPlate to give the final test concentrations.

Data Analysis

IonWorks data were either IC50 or EC50 value from one or more runs (Table 1). For each run, a noncumulative 8-point concentration-effect curve was produced and an IC50 or EC50 value was determined, with data for a given concentration of compound being from between 1 and 8 wells. When two or more runs were performed then the data were merged before fitting a Hill curve from which a single IC50 or EC50 value was derived. Data were normalized to vehicle (0.1% DMSO), and the differences between vehicle and top concentration tested were assessed for statistical significance using the Students t-test and showing greater than 25% change from control (being the amount needed to observe a difference beyond the experimental noise). Where an antagonistic effect was

observed with a compound, the data were then fitted to a simple pore block model using the Hill equation, allowing the Hill coefficient to vary but assuming that the compound would eventually cause complete block of the channel. Agonists were not considered in this study.

S1.2.2 GSK Screening Methods

IonWorks Barracuda: hERG and CaV1.2

Cell preparation

CaV1.2:

CaV1.2 (HEK293 $\alpha_{1C}/\beta_{2a}/\alpha_2\delta_1$) cells from Millipore were grown in DMEM-F12 with 10% FBS, Media was then supplemented with 400μg/mL G418, 100μg/mL Hygromycin B, 10mls/1Litre Pen/Strep and 0.625μg/mL Puromycin. Finally, the media was supplemented with the CaV1.2 blocker[4] at 10μM. All media was filter sterilised and handled using aseptic techniques.

Two T175cm^2 flasks of cells are taken from a tissue culture incubator. The media was aspirated and the cells briefly washed with 10mls warm PBS(-) to remove excess media before 3mL warm TrypLE was added incubated at 37℃ for 3 minutes. The flasks were tapped to dislodge cells, 10mls of external buffer (without Barium) was added and the suspension gently mixed. The volume was then transferred to 2 small falcon tubes (15mL) and centrifuged for 2 minutes at 1300 rpm. The supernatant was aspirate and the falcon tubes were re suspended in 6.5 mls of external buffer (without Barium) and then consolidated into 1 falcon tube and centrifuged for a further 2 minutes at 1300 rpm. The supernatant was aspirate and the cell pellet re suspended in 5 mls of external buffer containing Barium (warmed to 37℃) to give 3–4 $\times$ 10^6 cells/ml suspension. The cell suspension was pipetted through a p200 tip on the end of a 5ml stripette once. The small falcon with cell suspension was then added to the IonWorks Barracuda™.

hERG:

Chinese hamster ovary (CHO) cells stably expressing hERG were cultured in DMEM F12 Hams medium, supplemented with 10% FBS and 400μg/ml Geneticin (Gillie et al., 2013). Frozen cells were thawed in T175 flasks at 6–8 $\times$ 10^6 cells per flask, maintained at 37℃ in a humidified environment containing 5% CO_2 for 3–4 hours and then transferred to a 30℃ incubator containing 5% CO_2 and incubated for a further 72 hrs prior to assay. Cells with a confluence of over 80% were harvested by washing twice with warm PBS (without magnesium and calcium) and incubating at 37℃ with 5mls pre-warmed Versene for 6 minutes, followed by addition of 10mls of warm culture medium. The resulting suspension was transferred to a 15ml centrifuge tube and spun for 2 minutes at 1300 rpm. The supernatant was removed and cells were re-suspended in 5mls of warm culture medium and incubated at 37℃ for a further 5 minutes. The suspension was then centrifuged for 2 minutes at 1300rpm, the supernatant discarded and the pellet re-suspended to a cell concentration of 4–5 $\times$ 10^6 cells/ml. This suspension was transferred to the IonWorks Barracuda™.

Experimental Protocols

CaV1.2:

[4] A small proportion of the CaV1.2 channels will be open at resting membrane potential (window current), which can lead to cell death due to calcium toxicity. By adding a blocker to the media we rescue the cells from this effect. The majority of the blocker is washed out before the screening assay is performed, and any that remains should have a consistent effect between wells.

CaV1.2 currents were recorded before and after the addition of compound using a Molecular Devices IonWorks Barracuda™ automated electrophysiology instrument in Population Patch-Clamp mode. The intracellular solution contained the following: 140mM Potassium Chloride, 2mM $MgCl_2$, 20mM HEPES, pH 7.3 with KOH. Amphotericin-B solution was prepared as 50mg/ml stock solution in dimethylsulfoxide (DMSO) and diluted to a final working concentration of 0.1 mg/ml in intracellular solution. The external solution contained the following: 140mM Sodium Chloride, 2mM $MgCl_2$, 10mM HEPES, 10mM Barium Chloride, pH 7.4 with NaOH. Barium was used as the charge carrier in the assay.

The voltage pulse protocol applied pre- and post- compound addition was as follows: CaV1.2 currents were activated by 20msec pulse to 0mV from a holding potential of -70 mV over 5 pulses at 2Hz. The value exported for each well was the 5th pulse and the difference between current before compound addition and peak current after addition with a 5min compound incubation.

hERG:

hERG currents were recorded before and after the addition of compound using a Molecular Devices IonWorks Barracuda™ automated electrophysiology instrument in Population Patch-Clamp mode. The KCl intracellular solution contained the following: 140mM KCI, 1mM $MgCl_2$, 1mM $CaCl_2$, 20mM HEPES, pH 7.3 with KOH. Amphotericin-B solution was prepared as 50mg/ml stock solution in dimethylsulfoxide (DMSO) and diluted to a final working concentration of 0.1 mg/ml in intracellular solution. The external solution was D-PBS(-). The voltage pulse protocol applied pre- and post- compound addition was as follows: hERG currents were activated by a 5500 msec depolarising pulse to 20 mV from a holding potential of -80 mV. The cells were then repolarised to -50 mV for 1000 msec to generate large outward tail currents.

Data Analysis

IonWorks Barracuda™ recordings are population patch measurements in which the average of the current across many cells is determined. Hence the word 'observation' is used for a single concentration response curve. There could be several observations on a certain day.

CaV1.2:

Data were normalised to the high and low controls. Low controls were wells in which 100μM Cadmium Chloride was added for the CaV1.2 blocker assay. High controls were wells in which only DMSO was added for the CaV1.2 assay. The normalised data were analysed by using ActivityBase software. The amount of block was determined from the maximum peak response normalised to the base line. Concentration response data were derived using a four parameter logistic fitting procedure. pIC_{50} values were determined from these inhibition curves.

hERG:

Data were normalised to high and low controls. Low control wells contained 50μM Quinidine and high control wells contained equivalent quantities of DMSO. The normalised data were analysed by using ActivityBase software. The amount of tonic block was calculated from the peak tail current (the maximum amplitude of the tail current obtained after the voltage step to -50 mV). Concentration response data were derived using a four parameter logistic fitting procedure. pIC_{50} values were determined from these inhibition curves.

IonWorks Quattro: NaV1.5 and KCNQ1

Cell preparation

Human NaV1.5:

Human embryonic kidney-293 (HEK293) cells from Millipore were stably transfected with human NaV1.5 expression vector (pCIN5-hNaV1.5). Cells were cultured in DMEM with F12, supplemented with 10% FBS, 1x NEAA, plus 400μg/ml geneticin. Cells were grown and maintained at 37℃ in a humidified environment containing 5% CO_2. Media without geneticin was used for cell harvesting. Cells with less than 80% confluency were detached from the T75 culture flask for passage and harvesting using TrypLE or Versene. After media aspiration cells were washed with pre-warmed Ca^{2+}- and Mg^{2+}-free D-PBS. Then 3 ml pre-warmed TrypLE or Versene were added for 3–5 min, respectively, followed by addition of 10–12 ml pre-warmed Ca^{2+}- and Mg^{2+}-containing D-PBS. Finally cells were gently mixed 3–4 times. The suspension was centrifuged at 300 x G for 2 minutes, the pellet resuspended to a cell concentration of of 2–3 million cells/ml and that solution added to the IonWorks™ instrument.

KCNQ1:

Chinese hamster ovary (CHO) cells from Millipore were stably transfected with KCNQ1 (also known as Kv1.7 or KvLQT1) — the pore forming unit of the cardiac potassium current inward rectifier, and KCNE1 (also known as minK) the auxiliary subunit in the cardiac ion channel. Cells were cultured in IMDM ISCOVE media, supplemented with 10% FBS and 800μg/ml geneticin, 1ml hygromycin, 5ml Pen/Strep and filtered. Cells were thawed in T175 flasks at 6–8 million cells per T175 flask, maintained at 37℃ in a humidified environment containing 5% CO_2 for 24 hours and transferred to a 30℃ incubator containing 5% CO_2 and incubated for another 48 hrs before assaying. On day of assay, confluency of cells should be $< 40\%$ for screening. Media were removed, and cells were washed with warm KCNQ1 external solution (without magnesium and calcium). 3 ml pre-warmed TrypLE was added for 2–3 mins, followed by addition of 10 ml of warm KCNQ1 external solution (without magnesium and calcium). The suspension was placed into a 15 ml centrifuge tube and spun for 2 mins at 1K rpm. The supernatant was removed and cells re-suspended in 5 ml of warm KCNQ1 external solution (with magnesium and calcium). The pellet was re-suspended to a cell concentration of 3.5–4.5 million cells/ml and that solution added to the IonWorks™.

Experimental Protocols

All currents were recorded before and after the addition of compound using a Molecular Devices IonWorks Quattro automated electrophysiology instrument in Population Patch-Clamp mode.

Human NaV1.5:

The intracellular solution contained the following: 100mM K-gluconate, 40mM KCl, 3.2mM $MgCl_2$, 5mM HEPES, 3mM EGTA, pH 7.3 with KOH. Amphotericin-B solution was prepared as 50mg/ml stock solution in di-methylsulfoxide (DMSO) and diluted to a final working concentration of 0.1 mg/ml in intracellular solution. The external solution was D-PBS and contained the following: 0.90mM $CaCl_2$, 2.67mM KCl, 1.47mM KH_2PO_4, 0.493mM $MgCl_2$, 137.9mM NaCl, 8.06mM Na_2HPO_4, pH 7.4. All wells with a pre- and post-drug addition resistance of $> 20\text{M}\Omega$ and which yielded a > 200pA transient inward NaV current were included in the analysis.

The voltage pulse protocol applied pre- and post- compound addition was as follows: From a holding potential of -80mV (30 seconds), a train of five 200 millisecond depolarising voltage pulses were applied at a frequency of 2 Hz. The peak of the inward currents during the first and fifth 0 mV depolarisation were exported for the pre- and post-drug conditions. The amount of compound block observed at the fifth pulse determines the accumulated block observed and is expressed a percentage

of the pre-compound current observed at the first pulse to give a measure of the "global" (tonic and use-dependent) block achieved by the compound.

KCNQ1:

The KCNQ1 internal solution contained the following: 100mM Potassium Gluconate, 54mM Potassium Chloride, 3.2mM $MgCl_2$, 5mM HEPES, pH 7.3 with KOH. All solutions were filtered before use. Amphotericin-B solution was prepared as 50mg/ml stock solution in dimethylsulfoxide (DMSO) and diluted to a final working concentration of 0.1mg/ml in intracellular solution. The KCNQ1 external solution with Ca^{2+} and Mg^{2+} contained the following: 65mM Sodium Gluconate, 70mM Sodium Chloride, 5mM Potassium Chloride, 0.5mM $MgCl_2$, 1mM $CaCl_2$, 5mM HEPES, pH 7.4 with NaOH. The KCNQl external solution without Ca^{2+} and Mg^{2+} contained the following: 65mM Sodium Gluconate, 70mM Sodium Chloride, 5mM Potassium Chloride, 5mM HEPES, pH 7.4 with NaOH. The voltage pulse protocol applied pre- and post- compound addition was as follows: From a holding potential of -80mV (100ms), test opener potential 0 mV for 4s, step to -10mV for 2s, holding potential -80mV for 5s, test blocker potential $+50$mV for 4s, step to -10mV for 2s. The amount of compound block observed at the end of the $+50$mV pulse and is expressed a percentage of the pre-compound current observed at the beginning of the $+50$mV pulse to give a measure of the tonic block achieved by the compound.

Data Analysis

IonWorks™ Quattro recordings are population patch measurements in which the average of the current across many cells is determined. Hence the word observation is used for a single concentration response curve. There could be several observations on a certain day.

Human NaV1.5:

Comparisons between pre-drug peak transient inward currents at the first pulse and post-drug peak transient inward currents fifth pulse were used to determine the global inhibitory effect of the compound. Data were normalised to the high and low controls. Low controls were wells in which 100μM tetracaine was added for the NaV1.5 blocker assay. High controls were wells in which only 1% DMSO was added for the NaV1.5 blocker assay. The normalised data were analysed by using ActivityBase software. The amount of NaV1.5 current inhibition observed at the fifth depolarising pulse after compound addition was expressed as a percentage of the peak current observed at the first pulse before compound addition and used to generate a global block concentration dose response.

KCNQ1:

Data were normalised to the high and low controls. Low controls were wells in which an internal characterised full block compound was added for the KCNQ1 blocker assay. DMSO concentration in compound wells and high controls wells were only 0.25% for the KCNQ1 assay. The normalised data were analysed by using ActivityBase software. The current elicited at the end of the 50mV pulse is normalised to that at the start of the pulse and normalised to control data.

For all channels:

Concentration response data were derived using a four parameter concentration effect curve fitting procedure. pIC50 values and Hill coefficients were determined from these inhibition curves.

S1.3 Open source simulation code

All of the data and code used to perform the simulations presented here have been released as free open-source software under the BSD licence[5]. This licence gives minimal restrictions on re-use, whether in an academic or commercial setting.

The software is available to download from `http://www.cs.ox.ac.uk/chaste/download.html` - by clicking on the "Bolt-on projects" tab, and selecting the `Jptm2014Mirams` project. All code and data will be provided in a zipped archive format. To run the simulations you will need to download and install 'Chaste', the project is only guaranteed to be compatible with version 3.2 (released spring 2014).

Since the models are deterministic ODE systems, when the state variables take the same value at the start of a 'pace' and at the end (1 second later in this case), then the model must be in a "pseudo" steady state (limit cycle orbit). In practice this never happens, due to exponential decay towards this state, numerical method accuracy, and machine precision. So the 1Hz 'steady state' is defined as when the L_2 norm of the ODE state variables changes by less than an absolute value of 10^{-6} between the start of a pace and the end. In all situations we have encountered, this is a sufficient condition for the APD to change by less than 0.01 ms when running from this 'pseudo-steady state' to a 'reference steady state' evaluated at 100,000 paces.

We have also made use of a meta-model (model of the simulation result) for very fast evaluation of the credible regions, shown shaded on (e.g.) Figure S1, which would otherwise take thousands of simulations to evaluate. In this case we directly evaluate the simulated action potential duration for around a large number of possible combinations of ion channel block on the 5 channels of interest, with each ranging from 0–100% block. The spread parameters σ for the logistic distribution of pIC50 values that were used to infer possible input screening values (as described in Elkins et al. (2013)) are given in Table S1, having been measured from logistic distributions fitted to multiply repeated positive controls.

Table S1: Variability parameters for the Quattro and Barracuda screens at AZ and GSK. The Q dataset simulations use the same parameters as Elkins et al. (2013) (reproduced here for completeness), and the B&Q2 dataset uses the newly evaluated parameters below. Note that the variability on hERG in the M&Q dataset was assumed to be the same as Q (as it would be very difficult to quantify).

Channel	pIC50 spread parameter (σ)	
	Q	B&Q2
hERG	0.103 (Q)	0.146 (B)
CaV1.2	0.160 (Q)	0.120 (B)
NaV1.5	0.076 (Q)	0.139 (Q)
KCNQ1	0.140 (Q)	0.140 (Q)
Kv4.3	0.086 (Q)	–

This five-dimensional parameter sweep is used to store the action potential duration at around a million points throughout this 5d hyper-cube, and then simple linear interpolation is used to quickly "look up" the action potential duration at any point in this space. See the `ParameterBox`,

[5]details of the BSD licence can be found at `http://opensource.org/licenses/BSD-3-Clause`

LookupTableGenerator, and LookupTableReader and associated test classes in the ApPredict project for details of this. It took around a month on a 12-core machine to generate a 1.5–2 million point lookup table for each of these models.

By estimating the effect at each point before evaluating it directly, we get an estimate for the error there may be in the predictions we make. As the space becomes more refined, we can show that the vast majority of predictions are associated with less than 1ms error compared to a direct evaluation. This is not the case at 'bifurcations' (for example where the O'Hara model goes from repolarising to not repolarising under hERG block), but we believe the majority of predictions are in 'well-behaved' space since the directly evaluated APDs are continuous in concentration in all cases (see solid lines in the figures of section S1.1). We will be investigating ways to make these results available, perhaps via a web-service, to remove the need for each user to recreate them.

S1.4 TQT study concentration calculations

These are given in a separate spreadsheet Supplementary Material S2, which contains the necessary working for calculating maximum free plasma concentration estimates from maximum plasma concentration (C_{max}), percent plasma binding (PPB) and molecular weight using the following relationships:

$$1000 \times \frac{C_{max} \text{ [in ng/mL]}}{\text{Molecular Weight [in g/mol]}} = C_{max} \text{ [in nM]},$$

and

$$\text{Maximum free plasma concentation} = C_{max} \times (100 - \text{PPB})\,.$$

S1.5 Full contingency table results

There are different results for detection of prolongation for each model, platform, and considering each concentration range (at or within 10x, 100x of the estimated TQT concentration). The results are shown in full in Tables S2–S10.

Table S2: A contingency table for O'Hara et al. (2011) predictions based on the IonWorks Quattro dataset. A match is defined as agreement at estimated TQT concentration (top table), within 10-fold (middle), or within 100-fold (bottom).

1-fold conc.		Thorough QTc change			
		$\geqslant$ 5ms	< 5ms	Totals	
Simulated 1Hz APD change	$\geqslant$ 5ms	2	1	3	Positive Predictive Value 67%
	< 5ms	12	19	31	Negative Predictive Value 61%
	Totals	14	20	34	
		Sensitivity 14 %	Specificity 95%		Accuracy 62%

10-fold conc.		Thorough QTc change			
		$\geqslant$ 5ms	< 5ms	Totals	
Simulated 1Hz APD change	$\geqslant$ 5ms	6	0	6	Positive Predictive Value 100%
	< 5ms	8	20	28	Negative Predictive Value 71%
	Totals	14	20	34	
		Sensitivity 43 %	Specificity 100%		Accuracy 76%

100-fold conc.		Thorough QTc change			
		$\geqslant$ 5ms	< 5ms	Totals	
Simulated 1Hz APD change	$\geqslant$ 5ms	10	0	10	Positive Predictive Value 100%
	< 5ms	4	20	24	Negative Predictive Value 83%
	Totals	14	20	34	
		Sensitivity 71 %	Specificity 100%		Accuracy 88%

Table S3: A contingency table for O'Hara et al. (2011) predictions based on the IonWorks Quattro with Barracuda hERG & CaV1.2 dataset. A match is defined as agreement at estimated TQT concentration (top table), within 10-fold (middle), or within 100-fold (bottom).

1-fold conc.		Thorough QTc change $\geqslant$ 5ms	< 5ms	Totals	
Simulated 1Hz APD change	$\geqslant$ 5ms	1	1	2	Positive Predictive Value 50%
	< 5ms	12	12	24	Negative Predictive Value 50%
	Totals	13	13	26	
		Sensitivity 8 %	Specificity 92%		Accuracy 50%

10-fold conc.		Thorough QTc change $\geqslant$ 5ms	< 5ms	Totals	
Simulated 1Hz APD change	$\geqslant$ 5ms	4	0	4	Positive Predictive Value 100%
	< 5ms	9	13	22	Negative Predictive Value 59%
	Totals	13	13	26	
		Sensitivity 31 %	Specificity 100%		Accuracy 65%

100-fold conc.		Thorough QTc change $\geqslant$ 5ms	< 5ms	Totals	
Simulated 1Hz APD change	$\geqslant$ 5ms	8	0	8	Positive Predictive Value 100%
	< 5ms	5	13	18	Negative Predictive Value 72%
	Totals	13	13	26	
		Sensitivity 62 %	Specificity 100%		Accuracy 81%

Table S4: A contingency table for O'Hara et al. (2011) predictions based on the IonWorks Quattro with manual hERG dataset. A match is defined as agreement at estimated TQT concentration (top table), within 10-fold (middle), or within 100-fold (bottom).

1-fold conc.		Thorough QTc change $\geqslant$ 5ms	< 5ms	Totals	
Simulated 1Hz APD change	$\geqslant$ 5ms	7	3	10	Positive Predictive Value 70%
	< 5ms	7	17	24	Negative Predictive Value 71%
	Totals	14	20	34	
		Sensitivity 50 %	Specificity 85%		Accuracy 71%

10-fold conc.		Thorough QTc change $\geqslant$ 5ms	< 5ms	Totals	
Simulated 1Hz APD change	$\geqslant$ 5ms	11	0	11	Positive Predictive Value 100%
	< 5ms	3	20	23	Negative Predictive Value 87%
	Totals	14	20	34	
		Sensitivity 79 %	Specificity 100%		Accuracy 91%

100-fold conc.		Thorough QTc change $\geqslant$ 5ms	< 5ms	Totals	
Simulated 1Hz APD change	$\geqslant$ 5ms	11	0	11	Positive Predictive Value 100%
	< 5ms	3	20	23	Negative Predictive Value 87%
	Totals	14	20	34	
		Sensitivity 79 %	Specificity 100%		Accuracy 91%

Table S5: A contingency table for ten Tusscher and Panfilov (2006) predictions based on the IonWorks Quattro dataset. A match is defined as agreement at estimated TQT concentration (top table), within 10-fold (middle), or within 100-fold (bottom).

1-fold conc.		Thorough QTc change $\geqslant$ 5ms	$<$ 5ms	Totals	
Simulated 1Hz APD change	$\geqslant$ 5ms	0	3	3	Positive Predictive Value 0%
	$<$ 5ms	14	17	31	Negative Predictive Value 55%
	Totals	14	20	34	
		Sensitivity 0 %	Specificity 85%		Accuracy 50%

10-fold conc.		Thorough QTc change $\geqslant$ 5ms	$<$ 5ms	Totals	
Simulated 1Hz APD change	$\geqslant$ 5ms	4	0	4	Positive Predictive Value 100%
	$<$ 5ms	10	20	30	Negative Predictive Value 67%
	Totals	14	20	34	
		Sensitivity 29 %	Specificity 100%		Accuracy 71%

100-fold conc.		Thorough QTc change $\geqslant$ 5ms	$<$ 5ms	Totals	
Simulated 1Hz APD change	$\geqslant$ 5ms	7	0	7	Positive Predictive Value 100%
	$<$ 5ms	7	20	27	Negative Predictive Value 74%
	Totals	14	20	34	
		Sensitivity 50 %	Specificity 100%		Accuracy 79%

Table S6: A contingency table for ten Tusscher and Panfilov (2006) predictions based on the IonWorks Quattro with Barracuda hERG & CaV1.2 dataset. A match is defined as agreement at estimated TQT concentration (top table), within 10-fold (middle), or within 100-fold (bottom).

1-fold conc.		Thorough QTc change $\geqslant$ 5ms	Thorough QTc change < 5ms	Totals	
Simulated 1Hz APD change	$\geqslant$ 5ms	0	1	1	Positive Predictive Value 0%
	< 5ms	13	12	25	Negative Predictive Value 48%
	Totals	13	13	26	
		Sensitivity 0 %	Specificity 92%		Accuracy 46%

10-fold conc.		Thorough QTc change $\geqslant$ 5ms	Thorough QTc change < 5ms	Totals	
Simulated 1Hz APD change	$\geqslant$ 5ms	4	0	4	Positive Predictive Value 100%
	< 5ms	9	13	22	Negative Predictive Value 59%
	Totals	13	13	26	
		Sensitivity 31 %	Specificity 100%		Accuracy 65%

100-fold conc.		Thorough QTc change $\geqslant$ 5ms	Thorough QTc change < 5ms	Totals	
Simulated 1Hz APD change	$\geqslant$ 5ms	7	0	7	Positive Predictive Value 100%
	< 5ms	6	13	19	Negative Predictive Value 68%
	Totals	13	13	26	
		Sensitivity 54 %	Specificity 100%		Accuracy 77%

Table S7: A contingency table for ten Tusscher and Panfilov (2006) predictions based on the IonWorks Quattro with manual hERG dataset. A match is defined as agreement at estimated TQT concentration (top table), within 10-fold (middle), or within 100-fold (bottom).

1-fold conc.		Thorough QTc change			
		$\geqslant$ 5ms	< 5ms	Totals	
Simulated 1Hz APD change	$\geqslant$ 5ms	2	4	6	Positive Predictive Value 33%
	< 5ms	12	16	28	Negative Predictive Value 57%
	Totals	14	20	34	
		Sensitivity 14 %	Specificity 80%		Accuracy 53%

10-fold conc.		Thorough QTc change			
		$\geqslant$ 5ms	< 5ms	Totals	
Simulated 1Hz APD change	$\geqslant$ 5ms	9	0	9	Positive Predictive Value 100%
	< 5ms	5	20	25	Negative Predictive Value 80%
	Totals	14	20	34	
		Sensitivity 64 %	Specificity 100%		Accuracy 85%

100-fold conc.		Thorough QTc change			
		$\geqslant$ 5ms	< 5ms	Totals	
Simulated 1Hz APD change	$\geqslant$ 5ms	11	0	11	Positive Predictive Value 100%
	< 5ms	3	20	23	Negative Predictive Value 87%
	Totals	14	20	34	
		Sensitivity 79 %	Specificity 100%		Accuracy 91%

Table S8: A contingency table for Grandi et al. (2010) predictions based on the IonWorks Quattro dataset. A match is defined as agreement at estimated TQT concentration (top table), within 10-fold (middle), or within 100-fold (bottom).

1-fold conc.		Thorough QTc change $\geqslant$ 5ms	Thorough QTc change $<$ 5ms	Totals	
Simulated 1Hz APD change	$\geqslant$ 5ms	0	0	0	Positive Predictive Value NaN%
	$<$ 5ms	14	20	34	Negative Predictive Value 59%
	Totals	14	20	34	
		Sensitivity 0 %	Specificity 100%		Accuracy 59%

10-fold conc.		Thorough QTc change $\geqslant$ 5ms	Thorough QTc change $<$ 5ms	Totals	
Simulated 1Hz APD change	$\geqslant$ 5ms	3	0	3	Positive Predictive Value 100%
	$<$ 5ms	11	20	31	Negative Predictive Value 65%
	Totals	14	20	34	
		Sensitivity 21 %	Specificity 100%		Accuracy 68%

100-fold conc.		Thorough QTc change $\geqslant$ 5ms	Thorough QTc change $<$ 5ms	Totals	
Simulated 1Hz APD change	$\geqslant$ 5ms	4	0	4	Positive Predictive Value 100%
	$<$ 5ms	10	20	30	Negative Predictive Value 67%
	Totals	14	20	34	
		Sensitivity 29 %	Specificity 100%		Accuracy 71%

Table S9: A contingency table for Grandi et al. (2010) predictions based on the IonWorks Quattro with Barracuda hERG & CaV1.2 dataset. A match is defined as agreement at estimated TQT concentration (top table), within 10-fold (middle), or within 100-fold (bottom).

1-fold conc.		Thorough QTc change			
		$\geqslant$ 5ms	< 5ms	Totals	
Simulated 1Hz APD change	$\geqslant$ 5ms	0	1	1	Positive Predictive Value 0%
	< 5ms	13	12	25	Negative Predictive Value 48%
	Totals	13	13	26	
		Sensitivity 0 %	Specificity 92%		Accuracy 46%

10-fold conc.		Thorough QTc change			
		$\geqslant$ 5ms	< 5ms	Totals	
Simulated 1Hz APD change	$\geqslant$ 5ms	3	0	3	Positive Predictive Value 100%
	< 5ms	10	13	23	Negative Predictive Value 57%
	Totals	13	13	26	
		Sensitivity 23 %	Specificity 100%		Accuracy 62%

100-fold conc.		Thorough QTc change			
		$\geqslant$ 5ms	< 5ms	Totals	
Simulated 1Hz APD change	$\geqslant$ 5ms	4	0	4	Positive Predictive Value 100%
	< 5ms	9	13	22	Negative Predictive Value 59%
	Totals	13	13	26	
		Sensitivity 31 %	Specificity 100%		Accuracy 65%

Table S10: A contingency table for Grandi et al. (2010) predictions based on the IonWorks Quattro with manual hERG dataset. A match is defined as agreement at estimated TQT concentration (top table), within 10-fold (middle), or within 100-fold (bottom).

1-fold conc.		Thorough QTc change $\geqslant 5$ms	Thorough QTc change < 5ms	Totals	
Simulated 1Hz APD change	$\geqslant 5$ms	2	2	4	Positive Predictive Value 50%
	< 5ms	12	18	30	Negative Predictive Value 60%
	Totals	14	20	34	
		Sensitivity 14 %	Specificity 90%		Accuracy 59%

10-fold conc.		Thorough QTc change $\geqslant 5$ms	Thorough QTc change < 5ms	Totals	
Simulated 1Hz APD change	$\geqslant 5$ms	9	0	9	Positive Predictive Value 100%
	< 5ms	5	20	25	Negative Predictive Value 80%
	Totals	14	20	34	
		Sensitivity 64 %	Specificity 100%		Accuracy 85%

100-fold conc.		Thorough QTc change $\geqslant 5$ms	Thorough QTc change < 5ms	Totals	
Simulated 1Hz APD change	$\geqslant 5$ms	10	0	10	Positive Predictive Value 100%
	< 5ms	4	20	24	Negative Predictive Value 83%
	Totals	14	20	34	
		Sensitivity 71 %	Specificity 100%		Accuracy 88%

References

Davies, M., Mistry, H., Hussein, L., Pollard, C., Valentin, J.-P., Swinton, J., Abi-Gerges, N., 2012. An in silico canine cardiac midmyocardial action potential duration model as a tool for early drug safety assessment. American Journal of Physiology - Heart and Circulatory Physiology 302 (7), H1466–H1480.

Elkins, R., Davies, M., Brough, S., Gavaghan, D., Cui, Y., Abi-Gerges, N., Mirams, G., 2013. Variability in high-throughput ion-channel screening data and consequences for cardiac safety assessment. Journal of Pharmacological and Toxicological Methods 68 (1), 112–122.

Gillie, D. J., Novick, S. J., Donovan, B. T., Payne, L. A., Townsend, C., 2013. Development of a high-throughput electrophysiological assay for the human ether-à-go-go related potassium channel hERG. Journal of Pharmacological and Toxicological Methods 67 (1), 33–44.

Grandi, E., Pasqualini, F., Bers, D., 2010. A novel computational model of the human ventricular action potential and Ca transient. J. Mol. Cellular Cardiology 48, 112–121.

O'Hara, T., Virág, L., Varró, A., Rudy, Y., 2011. Simulation of the undiseased human cardiac ventricular action potential: model formulation and experimental validation. PLoS computational biology 7 (5), e1002061.

Persson, F., Carlsson, L., Duker, G., 2005a. Blocking characteristics of hKv1.5 and hKv4.3/hKChIP2.2 after administration of the novel antiarrhythmic compound AZD7009. Journal of cardiovascular pharmacology 46 (1), 7–17.

Persson, F., Carlsson, L., Duker, G., Jacobson, I., 2005b. Blocking Characteristics of hERG, hNav1. 5, and hKvLQT1/hminK after Administration of the Novel Anti-Arrhythmic Compound AZD7009. Journal of cardiovascular electrophysiology 16 (3), 329–341.

ten Tusscher, K., Panfilov, A., 2006. Alternans and spiral breakup in a human ventricular tissue model. Am. J. Physiol. Heart Circ. Physiol. 291 (3), 1088–1100.
